# Serrated Leaf‐Like N‐Doped Copper Sulfide Enabling Bifunctional Oxygen Reduction/Evolution via Dual‐Mode Cathode Reactions for High Energy Density and Cycle Stability in Zinc–Air Batteries

**DOI:** 10.1002/advs.202413425

**Published:** 2025-04-04

**Authors:** Do Hwan Jung, Yong Hak Park, Dong Won Kim, Jong Hui Choi, Seungrae Cho, Keon‐Han Kim, Dong Gyu Park, Byungchan Han, Jeung Ku Kang

**Affiliations:** ^1^ Department of Materials Science and Engineering and NanoCentury Institute Korea Advanced Institute of Science and Technology (KAIST) 291 Daehak‐ro, Yuseong‐gu Daejeon 34141 Republic of Korea; ^2^ Department of Chemical and Biomolecular Engineering Yonsei University 50 Yonsei‐ro, Seodaemun‐gu Seoul 03722 Republic of Korea; ^3^ The Research Institute of Basic Science Seoul National University 1 Gwanak‐ro, Gwanak‐gu Seoul 08826 Republic of Korea

**Keywords:** bifunctional oxygen reduction/evolution reaction, dual‐mode cathode reactions, high energy density and cycle stability, serrated leaf‐like nitrogen‐doped copper sulfide, zinc–air battery

## Abstract

Zinc–air batteries (ZABs) are promising electrochemical energy storages, but inefficient oxygen reduction reaction (ORR) during discharging and oxygen evolution reaction (OER) during charging at their cathodes impede achieving high energy density and stable cycling. We report a serrated leaf‐like nitrogen‐doped copper sulfide (N‐CuS) cathode with conductive N 2p‐S 3p hybridized orbitals, oxygen‐transporting mesopores, and about fivefold larger surface area than Cu. A ZAB with the N‐CuS cathode exhibits a 788 mAh g^−1^ capacity (96% of theoretical) and a hitherto highest energy density of 916.0 Wh kg^−1^, surpassing one with the state‐of‐the‐art Pt/C+RuO₂ cathode (712.43 mAh g^−1^ and 874 Wh kg^−1^). Density functional theory calculations elucidate that O═O bond dissociation is 0.97 eV more favorable on N‐CuS than CuS. Subsequently, protonation of surface‐adsorbed *O to *OH occurs via dissociate (0.55 V), non‐spit associate (1.05 V), and split associate (1.05 V) pathways, with *OH then desorbing as OH^‐^. Under anaerobic conditions, copper oxide transitions from CuO to Cu_2_O (1.05 V) and eventually to Cu (0.75 V) releasing oxygen to sustain ORR. Additionally, a ZAB with the N‐CuS cathode achieves about threefold longer cyclability than one with the Pt/C+IrO₂ cathode, and about six‐fold longer cyclability than one with the Pt/C+RuO₂ cathode.

## Graphical abstract

A serrated leaf‐like nitrogen‐doped copper sulfide (N‐CuS) cathode with hybridized N 2p – S 3p orbitals is presented, mesopores for oxygen transport, and a five times larger electrochemical active surface area than Cu. In aerobic conditions, a zinc–air battery with N‐CuS achieves 788 mAh g⁻¹ and 916 Wh kg⁻¹, outperforming Pt/C+RuO₂. Under anaerobic conditions, CuO transitions to Cu₂O and Cu, sustaining oxygen reduction.

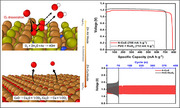

## 1 Introduction

Rechargeable electrochemical energy storage systems are essential for many applications across various fields, ranging from portable electronics to electric vehicles to large‐scale grid systems.^[^ ^1^ ^]^ Nowadays, lithium‐ion batteries (LIBs) dominate the energy storage industry,^[^ ^2^, ^3^, ^4^ ^]^ however, LIBs exhibit critical limitations, including safety concerns arising from the flammability and volatility of organic electrolytes,^[^ ^5^, ^6^ ^]^ as well as the finite and unevenly distributed reserves of lithium resources. Metal–air batteries present a promising alternative to address these limitations, offering significantly higher energy densities than conventional lithium‐ion batteries.^[^ ^6^, ^7^ ^]^ Metal anodes can be derived from s‐orbital alkali metals or d‐/p‐orbital alkaline earth and first‐row transition metals. While s‐orbital metals exhibit high reactivity, they are prone to surface passivation and pose significant safety risks due to spontaneous reactions in air or humid environments.^[^ ^8^ ^]^ Replacing s‐orbital metals with d‐ or p‐orbital metals addresses these issues, allowing the adoption of aqueous electrolytes, enhancing stability, and reducing costs. Among these, zinc (Zn) is particularly advantageous due to its earth abundance, low cost, and high theoretical capacity (820 mAh g^−1^),^[^ ^9^, ^10^ ^]^ which are critical for achieving the high energy densities desired in metal–air battery systems.^[^ ^11^ ^]^ Furthermore, the reliance of Zn–air batteries (ZABs) on oxygen directly extracted from the air as a reactant enables them to achieve significantly higher gravimetric and volumetric energy densities compared to lithium‐ion batteries (LIBs).^[^ ^12^, ^13^, ^14^, ^15^ ^]^ The limiting factor in this ZAB system is the catalytic activity of oxygen reduction reaction (ORR)/oxygen evolution reaction (OER).^[^ ^16^, ^17^, ^18^ ^]^ In the view of operation for ZABs, the OER benefits from lower overpotentials in the alkaline electrolytes compared to acidic conditions, while non‐precious metal catalysts exhibit higher stability and durability. Similarly, ORR in alkaline media supports an efficient four‐electron pathway, enabling high reaction efficiency and allowing cost‐effective non‐precious metal catalysts to perform comparably to platinum (Pt)‐based ones.^[^ ^19^ ^]^ Nevertheless, diffusion limitations and lower catalytic performance compared to acidic conditions with Pt‐based catalysts^[^ ^19^, ^20^ ^]^ remain challenges to address.

Copper (Cu) is one of the most abundant elements on Earth, and Zn–Cu batteries, also known as galvanic batteries, have been developed for centuries, typically using acidic and neutral electrolytes for primary batteries. However, when an alkaline electrolyte is used, the Zn–Cu battery can function as a secondary battery by utilizing its intrinsic standard reduction potential relative to Zn.^[^ ^21^ ^]^ These unique properties make Cu a promising cathode material when combined with secondary Zn battery systems, offering higher reversibility compared to Ag, Ni, Co, Bi, and their oxides/hydroxides.^[^ ^22^ ^]^ Furthermore, an alkaline electrolyte‐based rechargeable battery cell can be employed in an air‐dependent battery system to address operational challenges in low‐oxygen environments and enhance performance under ambient air conditions.^[^ ^23^ ^]^ This reaction pathway not only enhances catalytic activity but also contributes to higher energy conversion efficiencies in ZABs. However, the inherently low adsorption energy of oxygen species (*OH, *O, and *OOH) on Cu limits its catalytic activity, necessitating further Cu catalyst modification to enhance adsorption and improve reaction kinetics.

In this work, we synthesize a bifunctional serrated leaf‐like nitrogen‐doped copper sulfide (N‐CuS) directly deposited on copper foam cathode, that operates effectively in aerobic and anaerobic modes, contributing to high energy density and cycle stability in ZABs. Copper sulfide is a good candidate for air cathode catalyst due to its abundance, environmental benignity, cost‐effectiveness, and catalytic activity.^[^ ^24^ ^]^ Additionally, the high conductivity of copper sulfides^[^ ^25^ ^]^ and the copper substrate overall aid the facile electron transfer of the system.

A single‐step anodizing technique is developed and employed to boost an electrochemical surface area (ECSA) by lacerating the metal surface, resulting in the formation of copper sulfide nanoflakes that promote rapid oxygen transfer. Subsequently, nitrogen dopants are incorporated into copper sulfide nanoflake surfaces. The hybridization of N (2p) and S (3p) orbitals is exhibited to enable high electric conductivity, leading to significantly excellent ORR catalytic activity. Also doping of nitrogen alleviates the strong binding of oxygen intermediates to sulfur sites,^[^ ^26^ ^]^ enhancing the overall catalytic activity. Besides, the open mesoporous architecture of the N‐CuS ensures a steady supply of oxygen from the air. The N‐CuS cathode is exhibited to retain both metallic and oxidized coppers as redox‐active sites even in the absence of air. In aerobic conditions, the metallic sites function to convert oxygen to hydroxides, while sacrificing its copper oxides in anaerobic conditions. Moreover, we assemble the N‐CuS cathode with the Zn anode to construct a ZAB full cell (N‐CuS//Zn). Under aerobic conditions, the N‐CuS//Zn full cell is shown to achieve ultrahigh capacity and energy density exceeding those of a ZAB with the state‐of‐the‐art Pt/C + RuO_2_ cathode. On the other hand, under anaerobic conditions, copper oxides are exhibited to undergo cascade phase transitions, driving efficient ORR during discharging, whereas reverse transitions occur by oxygen generated via OER during charging. These cascade transitions, which are not typically observed in conventional ZABs, are demonstrated to enable extended operation in oxygen‐limited environments, such as deep underwater or at high altitudes. Additionally, cycle stability tests are performed to demonstrate that a ZAB with the N‐CuS cathode has prolonged cyclability.

## 2 Results and Discussion

**Figure 1:**
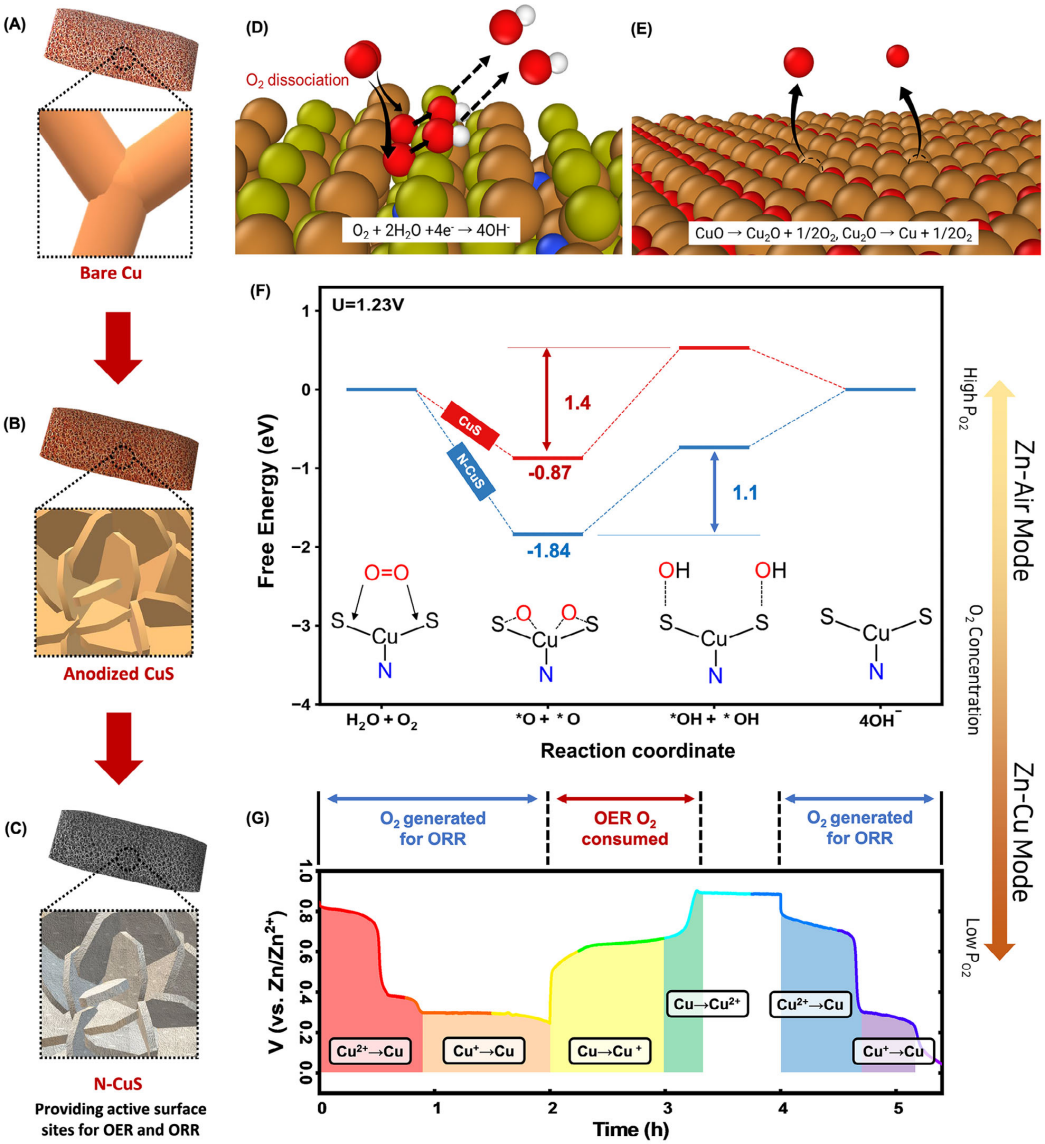
Schematic illustration of synthesis processes as well as Zn–air and Zn–Cu modes. Synthetic processes for A) bare Cu, B) anodized CuS, and C) N‐CuS cathode materials. D) Zn–air mode operating under aerobic conditions. E) Zn–Cu mode functioning under anaerobic conditions. F) Proposed reaction pathways for oxygen reduction reaction (ORR) and oxygen evolution reaction (OER) on the surface of N‐CuS in the Zn–air mode. G) Potential changes associated with copper oxide transitions (CuO → Cu_2_O → Cu) in the Zn–Cu mode.

The synthesis process, as depicted in **Figure** **1**A–C, employs anodization as a sophisticated electrochemical technique to create a redox‐active surface replete with defects. The electrically driven chemical reactions tailor the electrode surface according to the electrolyte used.^[^ ^27^ ^]^ In particular, sodium sulfide electrolyte facilitates the formation of a copper sulfide layer on the electrode surface after electrolysis. The as‐synthesized copper sulfide structure, illustrated in Figure 1B, exhibited a significant increase in ECSA. Furthermore, the incorporation of nitrogen dopants into the copper sulfide layer increased the ECSA by approximately 5 times compared to bare Cu foam (Figure 1C). Nitrogen dopants effectively mitigated the strong affinity of sulfur atoms for oxygen while simultaneously boosting electrical conductivity. Moreover, the N‐CuS electrode demonstrated the capability for dual‐mode cathodic reactions. Under aerobic conditions, it operated in an air‐cell mode, converting ambient oxygen to hydroxides, as described in Figure 1D. In contrast, under anaerobic conditions (Figure 1E), the cathode reactions enable the transformations of copper oxides into metallic copper, which generates oxygen necessary for ORR without relying on atmospheric oxygen. Figure 1F illustrates the dissociative ORR mechanisms on N‐CuS and CuS on the surface. ORR initiates with the cleavage of the O─O bond at the Cu─S bridge sites to form Cu─*O─S bonds, followed by protonation that results in the formation of *OH adsorbates relocated at S sites. The density functional theory (DFT) calculations elucidate that this ORR process occurs at an overpotential of 0.55 V, which is notably lower than 0.7 V on the CuS surface, resulting in faster kinetics than the CuS. Figure 1G shows the experimentally measured potential changes throughout charging and discharging cycles, where Cu^2+^ is reduced to Cu^+^ and subsequently to Cu^0^ for metallic Cu. These cascade transitions (CuO → Cu_2_O → Cu) during discharging result in the formation of O_2_ molecules, facilitating efficient ORR even under anaerobic conditions. This indicates that an N‐CuS electrode, when integrated into a gas purging cell, can sustain power generation even in environments with limited oxygen.

**Figure 2:**
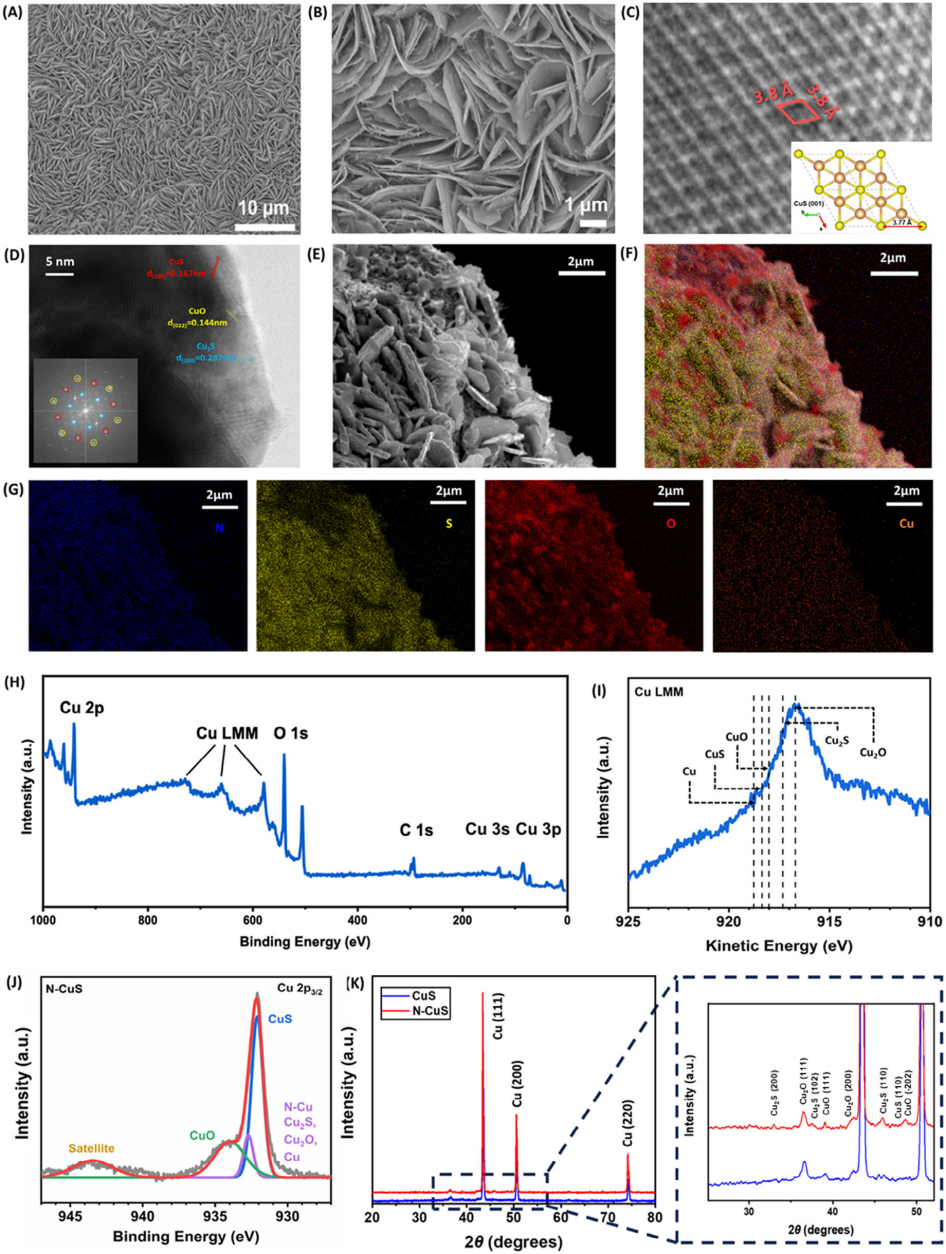
Structural characterizations of N‐CuS. (A and B) Low and high magnification of Field emission scanning electron microscopy (FESEM) image of the N‐CuS C) High‐resolution transmission electron microscopy (HRTEM) of N‐CuS and inset shows a covellite CuS. D) TEM image of the N‐CuS and inset shows fast Fourier transformation (FFT). E) and F) FESEM image and energy‐dispersive X‐ray spectroscopy (EDS) mapping overlay. G) elemental EDS mapping images for N, Cu, S, and O. H) X‐ray photoelectron spectroscopy (XPS) spectrum, I) Cu‐LMM Auger electron spectrum, and J) deconvoluted Cu 2p_3/2_ XPS spectrum of N‐CuS. K) X‐ray diffraction (XRD) spectra of as‐prepared CuS and N‐CuS.

Field‐emission scanning electron microscopy (FESEM) images were obtained to investigate the effects of anodizing and nitrogen‐doping processes on surface morphology. Figure S1 (Supporting Information) shows that CuS growth on Cu foam through anodization results in a densely packed flake‐like structure. This morphology increases the surface area‐to‐mass ratio, thereby enhancing the accessibility of redox carriers to the active sites. Through a detailed examination of the anodization process over time, the optimal conditions for high catalytic activity were determined. The subsequent incorporation of nitrogen dopants transformed these flakes into serrated leaf‐like structures, depicted in **Figure** **2**A,B, where each leaflet is a few micrometers long and approximately 50 nm thick. Additionally, high‐resolution transmission electron microscopy (HRTEM) images of pulverized N‐CuS were obtained to examine the shape, size, and lattice planes. As shown in Figure 2C, a distinctive d‐spacing of 3.8 Å was observed in N‐CuS, which well‐matched the distance between S atoms in the (001) plane of theoretically calculated covellite CuS (3.77 Å).^[^ ^28^ ^]^ Figure 2D and Figure S2 (Supporting Information) show different spots of N‐CuS, revealing the presence of CuS (200) with d‐spacing of 0.167 nm, Cu_2_S (200) with 0.287 nm, CuO (022) with 0.144 nm, and Cu_2_O (200) with 0.215 nm. These values were matched with the corresponding inset images of fast Fourier transform (FFT), respectively.^[^ ^29^, ^30^, ^31^, ^32^ ^]^ Figure 2E–G displays the FESEM and energy‐dispersive spectroscopy (EDS) mapping images, which demonstrate an even distribution of N, Cu, S, and O elements throughout the synthesized material. To further elucidate the surface chemical state, X‐ray photoelectron spectroscopy (XPS) and Auger electron spectroscopy (AES) analyses were performed, with the results shown in Figure 2H–J and Figure S3A,B (Supporting Information). The XPS survey in Figure 2H shows distinct copper 3s and 3p, Cu LMM, O 1s, and C 1s peaks, where the adventitious carbon peak ≈248.8 eV serves as the reference.^[^ ^33^ ^]^ The Cu‐LMM Auger electron spectra (Figure 2I) were also utilized since the copper oxidation states could not be discernible from Cu 2p spectra alone. The deconvoluted Cu 2p_3/2_ XPS spectrum of the N‐CuS indicates a mixture of Cu, CuS, and Cu₂S, as well as CuO, Cu₂O, and Cu─N (Figure 2J). Figure S3C,D (Supporting Information) showed a shift in the Cu 2p_3/2_ peak by 0.1 eV (from 932.0 to 932.1 eV), indicating the formation of a new chemical phase. Additionally, the increased intensity of the N‐doped Cu (N─Cu) peak of the N‐CuS compared to the CuS sample suggests the successful incorporation of N atoms via N₂ plasma treatment. In Figure S3E (Supporting Information), the presence of nitrogen is further confirmed by a peak at 398.7 eV, which can be deconvoluted into N─Cu (398.7 eV) and chemisorbed N (400.0 eV) peaks.^[^ ^34^ ^]^ Figure S3F (Supporting Information) presents asymmetric S 2p_1/2_ and 2p_3/2_ peaks at 163.1 and 162.0 eV, respectively, corresponding to the S^2^⁻ state in the CuS bond.^[^ ^35^, ^36^, ^37^ ^]^ Additionally, a peak at 165.6 eV signifies the S─S bond, while another peak at 169.0 eV is attributed to SO₄^2^⁻. Furthermore, X‐ray diffraction (XRD) patterns confirmed the crystalline structures of both CuS and N‐CuS. The XRD spectra (Figure 2K) feature prominent peaks corresponding to the copper metal planes, including Cu (111) at 2θ = 43.5°, Cu (200) at 2θ = 50.4°, and Cu (220) at 2θ = 74.4°.^[^ ^38^ ^]^ Peaks at 2θ = 32.3°, 37.4°, and 45.9° correspond to Cu_2_S (200), (102), and (110),^[^ ^39^, ^40^ ^]^ those at 36.4° and 42.5° correspond to Cu_2_O (111) and (200),^[^ ^41^ ^]^ and the peak at 45.6° corresponds to CuS (110),^[^ ^42^, ^43^ ^]^ and the peak at 39.1° and 48.7° correspond to CuO (111) and (‐202).^[^ ^44^ ^]^ Based on consistent XRD results of CuS and N‐CuS, the N dopants had negligible impact on the crystalline structure.

**Figure 3:**
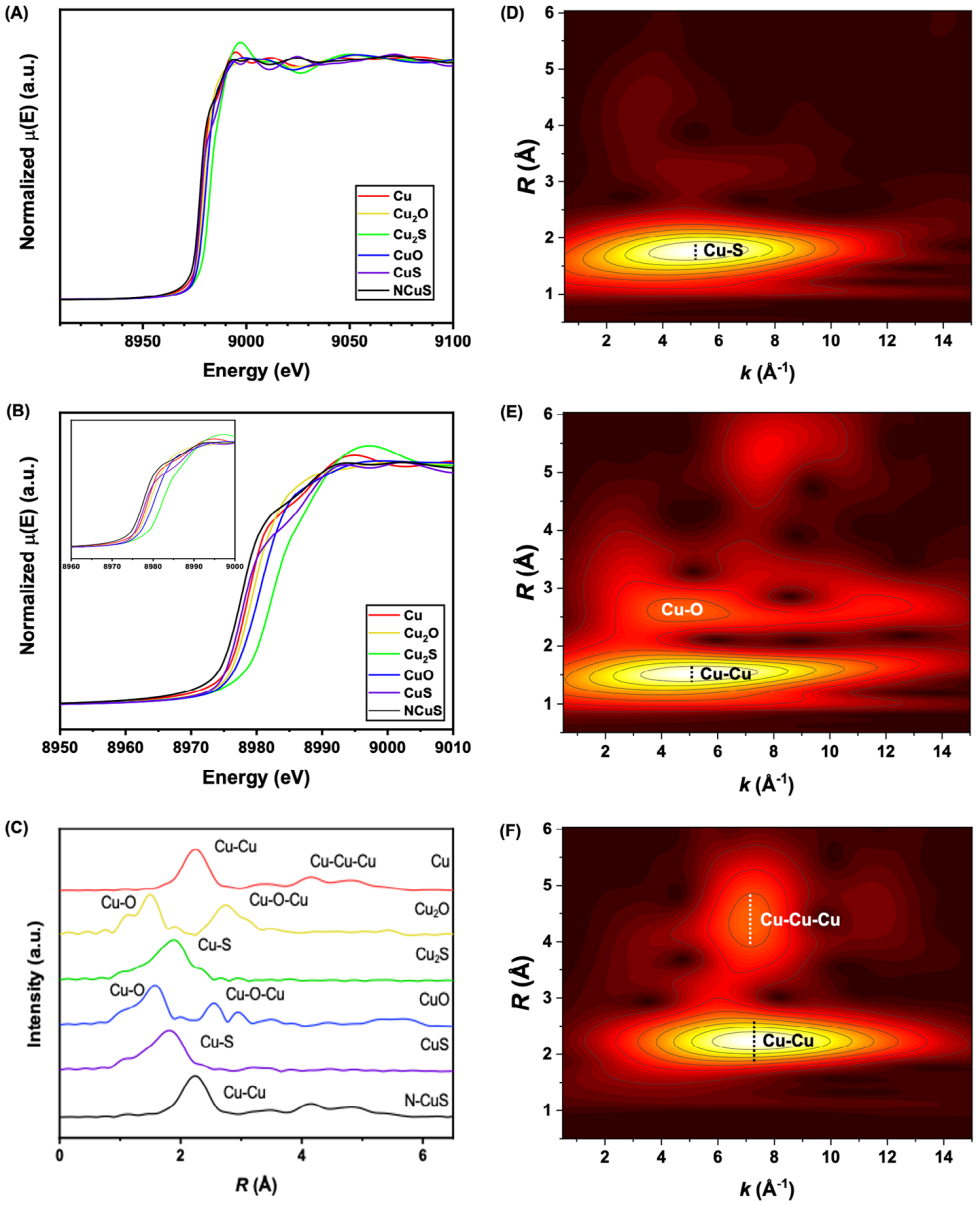
Synchrotron X‐ray absorption (XAS) spectra of N‐CuS. A) Cu K‐edge XAS spectra and B) X‐ray absorption near‐edge structure (XANES) spectra and their edges (inset) for N‐CuS and related reference species. C) Normalized *k* ^2^‐weighted Fourier Transform (FT)‐EXAFS spectra into *R*‐space. D), E), and F) 2D Morlet wavelet transform (WT) contours plot of *k* ^2^‐weighted spectra for CuS, CuO, and N‐CuS, respectively.

X‐ray absorption spectroscopy (XAS) analysis was conducted on the pulverized N‐CuS to clarify its atomic configurations and oxidation states. **Figure** **3**A presents the Cu K‐edge XAS spectra of N‐CuS, Cu foil, and copper compounds including Cu_2_O, CuO, CuS, and Cu_2_S. The X‐ray absorption near edge structure (XANES) spectra are revealed in Figure 3B. The N‐CuS spectrum (black line) has the onset energy (E_0_) of 8978.0 eV similar to 8977.8 eV of the copper foil and less than Cu^1+^ species – 8978.6 eV and 8979.6 eV for Cu_2_O and Cu_2_S, respectively. The edge area falls between Cu^1+^ and Cu^0^ with slight shouldering toward Cu^2+^ curves. This finding suggests that N‐CuS exhibits a mix of Cu^0^, similar to those found in the Cu foil, as well as states from both copper oxide and sulfide species.^[^ ^45^ ^]^ Figure 3C exhibits the normalized *k* ^2^‐weighted Fourier transform (FT)‐EXAFS spectra into *R*‐space, highlighting the prominent peaks from various scattering paths. The EXAFS spectrum of N‐CuS closely resembles that of the Cu foil, with a peak at 2.55 Å corresponding to the Cu─Cu bond distance, attributed to the Cu─Cu (1.1) single scattering path.^[^ ^46^ ^]^ The peaks within the range of 4 Å ≤ *ΔR* ≤ 5 Å are owing to both double and single scattering paths from more distant Cu atoms. Furthermore, the Morlet wavelet transforms (WTs), depicted in Figure 3D–F and Figure S4 (Supporting Information), were used to analyze the coordination environments of copper species within N‐CuS. The EXAFS spectra were correlated with the scattering paths of electrons, as detailed in Table S1 (Supporting Information). Figure 3D presents the 2D WT contour plot of CuS, featuring a distinct lobe near Δ*R* = 2.55 Å in Δ*k* = 0.5‐11 Å^−1^ region, attributed to the Cu─S (1.1) single scattering path. Similarly, the 2D WT contour plot of CuO (Figure 3E) reveals a primary lobe at Δ*R* = 1.85 Å in Δ*k* = 0.5‐13 Å^−1^ region, arising from Cu‐O single scattering. Additionally, a secondary diffuse lobe is linked to Cu─Cu single scattering and various multiple scattering paths. Comparable features were observed in Cu^1+^ species for both oxide and sulfide (Figure S4, Supporting Information). The 2D WT contour plot in *R*‐ and *k*‐spaces of N‐CuS (Figure 3F) reveals a primary lobe near Δ*R* = 2.55 Å in the Δ*k* = 2–14 Å^−1^ region, originating from Cu─Cu (1.1) interactions in the metal foam substrate. A secondary lobe, located between *ΔR* = 3.6‐5.1 Å and *Δk* = 5–9 Å^−1^, is associated with multiple scattering paths. Moreover, the DFT‐based formation energies of bulk copper sulfides were explored as a function of S composition. Bulk CuS in its covellite structure was found to be the most thermodynamically stable among possible copper sulfide structures, using face‐centered cubic (FCC) Cu and S_8_ solid as reference states (Figure S5A,B, Supporting Information). For the CuS surface, the (001) plane is identified as the most thermodynamically stable surface, according to the slab model.^[^ ^47^, ^48^ ^]^ The surface energies, which vary across different Miller indices and terminations due to the varying bonds that must be cleaved to create the surface, were calculated for three alternative terminations on the most stable (001) surface, as shown in Figure S5C (Supporting Information). The configurations for substitutional and interstitial doped structures are shown in Figure S5D (Supporting Information), and the energies for each doping are detailed in Table S2 (Supporting Information). Such analysis was used to determine the most stable configuration, revealing that N‐interstitial doping is energetically more favorable than N‐substitutional doping.

**Figure 4:**
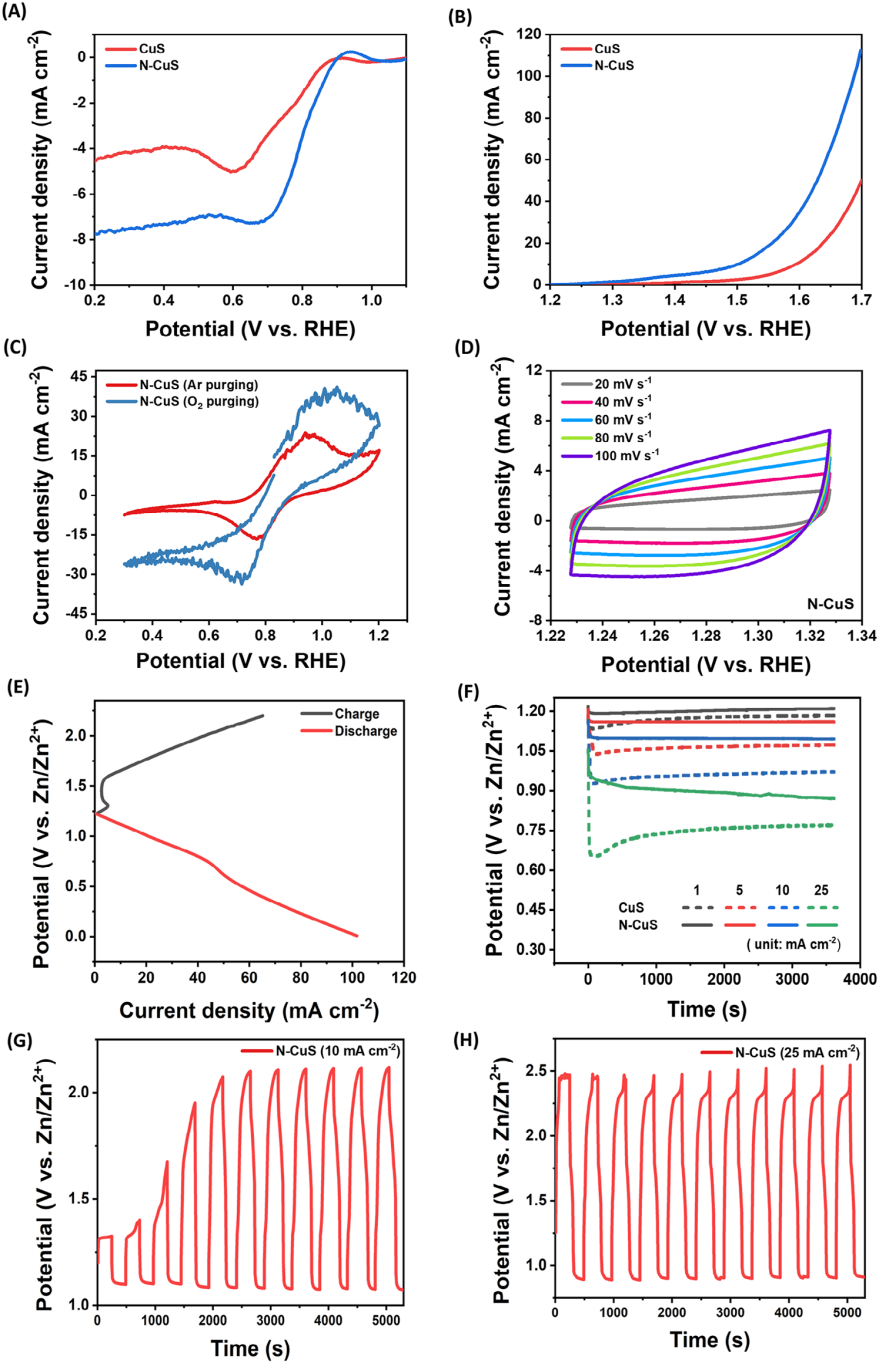
Electrochemical characterizations of N‐CuS as the cathode. A) linear sweep voltammetry (LSV) analysis of CuS and N‐CuS with O_2_ purging condition for ORR. B) Corresponding LSV for OER. C) Cyclic voltammetry (CV) analysis of CuS and N‐CuS for ORR/OER. D) CV plots for N‐CuS at the scan rates of 20, 40, 60, 80, and 100 mV s^−1^. E) Charging‐discharging profile of an O_2_ purging cell with N‐CuS. F) Chronopotentiometry at 1, 5, 10, and 25 mA cm^−2^ discharging rates. Galvanostatic cycling with potential limitation (GCPL) analysis at the discharging rates of G) 10 mA cm^−2^ and H) 25 mA cm^−2^.

The electrochemical performance of N‐CuS was characterized using an H‐type cell with a three‐electrode configuration to investigate its half‐cell properties, where N‐CuS served as the working electrode, the Hg/HgO electrode as the reference electrode, and the Pt electrode as the counter electrode in a 1 m KOH electrolyte. The ORR performance of N‐CuS was evaluated by linear sweep voltammetry (LSV), with CuS, bare Cu foam, and Pt/C+RuO_2_ on Cu foam used as control samples. **Figures** **4**A and S6A (Supporting Information) show that the N‐CuS has a half‐wave potential in ORR (E_ORR,1/2_) of 0.790 V, comparable to Pt/C+RuO_2_ (0.754 V) with Pt/C on Cu foam giving 0.845 V, CuS (0.760 V), and bare Cu foam (0.407 V). N‐CuS demonstrated the highest limiting current density of 7.724 mA cm^−2^, surpassing Pt/C+RuO_2_ (6.717 mA cm^−2^) and CuS (4.580 mA cm^−2^). Moreover, N‐CuS and CuS showed relatively steady currents below 0.65 V versus RHE, whereas Cu foam and noble metal catalyst coated on Cu foam exhibited two distinctive cathodic peaks between 0.4 and 0.8 V versus RHE and sharply increased the current densities below 0.4 V versus RHE. Notably, the two peaks resulting from the reduction of surface‐oxidized species and the sharply increased current may play a role in the formation of oxygen peroxide species.^[^ ^49^, ^50^ ^]^ Figure 4B and Figure S6B (Supporting Information) display the OER polarization curves. At the current density of 10 mA cm^−2^ (E_OER,j = 10_), N‐CuS exhibited a low overpotential of 271 mV, lower than those of comparison samples which were coated onto Cu foam such as Pt/C+RuO_2_ (398 mV), RuO_2_ (348 mV), CuS (365 mV), and Cu (419 mV). The potential difference between E_OER,j = 10_ and E_ORR,1/2_ was determined to evaluate the activity of the bifunctional oxygen electrocatalyst. N‐CuS exhibited a much smaller potential difference (0.711 V) than commercial Pt/C+RuO_2_ (0.876 V) and recently reported bifunctional catalyst (Table S3, Supporting Information).^[^ ^51^, ^52^, ^53^, ^54^, ^55^, ^56^ ^]^ Such high activities were attributed to the redox‐active properties of N‐CuS. Figure S7 (Supporting Information) displays the ex‐situ XPS of N‐CuS after ORR at 0.2 V versus RHE and OER at 1.7 V versus RHE. All elements were well observed in the survey scan (Figure S7 A,B, Supporting Information). The CuS‐induced peaks decreased, and distinct changes were observed in the Cu 2p_3/2_ spectra. The dominant peak after the OER test corresponded primarily to CuO, whereas a more reduced species appeared after the ORR test (Figure S7C,D, Supporting Information). The changes in intensity and shapes of the N 1s and S 2p peaks after the reaction are depicted in Figure S7E–H (Supporting Information) and the values were summarized in Table S4 (Supporting Information). In addition, the stability of N‐CuS and Pt/C+RuO₂ was evaluated for ORR at 0.6 V versus RHE and OER at 1.6 V versus RHE (Figure S8, Supporting Information). N‐CuS exhibited excellent stability, retaining 81.27% of its current for ORR and 61.05% for OER over 20 h, whereas Pt/C+RuO₂ showed a significant decline in activity, down to 29.30% for ORR and 32.78% for OER. The ORR/OER catalytic activity of N‐CuS was confirmed under different purging conditions (Ar and O_2_), as illustrated in Figure 4C. Additionally, the ECSA was estimated using multiple CV measurements at various scan rates (20, 40, 60, 80, and 100 mV s^−1^) to determine double layer capacitance (C_dl_), as shown in Figure 4D and Figure S9A,B (Supporting Information). The C_dl_ was calculated from the slope of the linear lines obtained by plotting the average current density from anodic and cathodic scans against scan rates at 1.28 V versus RHE. Figure S9C (Supporting Information) displays that the C_dl_ (41.4 mF cm^−2^) for the N‐CuS sample is significantly higher than that (8.2 mF cm^−2^) for bare Cu foam, ensuring fast kinetics of N‐CuS during electrochemical reactions. Further testing in a gas purging cell configuration was conducted under a constant oxygen flow to evaluate characteristics (Figure 4E), the graphic images of the detailed cell configuration were shown in Figure S10 (Supporting information), in which Zn metal, N‐CuS, and 6 m KOH were employed as the anode, cathode, and electrolyte, respectively. ORR performance was assessed through charging/discharging polarization curves via LSV. Cell potentials were evaluated under various discharging current densities (1, 5, 10, and 25 mA cm^−2^) to validate the effect of nitrogen doping (Figure 4F). The maximum discharging efficiency was achieved for N‐CuS following 4 min of N₂ plasma treatment (Figure S11, Supporting Information), displaying high‐voltage plateaus of 1.20, 1.16, 1.09, and 0.87 V at current densities of 1, 5, 10, and 25 mA cm^−2^, respectively. In contrast, CuS exhibited lower plateaus of 1.17, 1.05, 0.93, and 0.75 V under the same conditions. Cyclability was also evaluated through galvanostatic cycling with potential limitation (GCPL) at 10 and 25 mA cm^−2^ for 4 minutes per cycle (Figure 4G,H). N‐CuS exhibited a lower charging voltage and a higher discharging voltage compared to CuS, indicating that the cell does not require high voltages to maintain superior discharging characteristics. Moreover, the discharge rate performance of N‐CuS and Pt/C on Cu foam was evaluated by applying 1, 5, 10, 50, and 100 mA cm^−2^ (Figure S12, Supporting Information). The voltage obtained for N‐CuS closely matched that of Pt/C on Cu foam at relatively low current densities. However, at a current density of 100 mA cm^2^, N‐CuS exhibited a higher voltage of 0.69 V, compared to 0.60 V for Pt/C on Cu foam. In addition, the ZAB with the N‐CuS cathode showed a maximum power density of 101 mW cm^−2^ (Figure S13A, Supporting Information) and lasted more than 1000 cycles (Figure S13B, Supporting Information).

**Figure 5:**
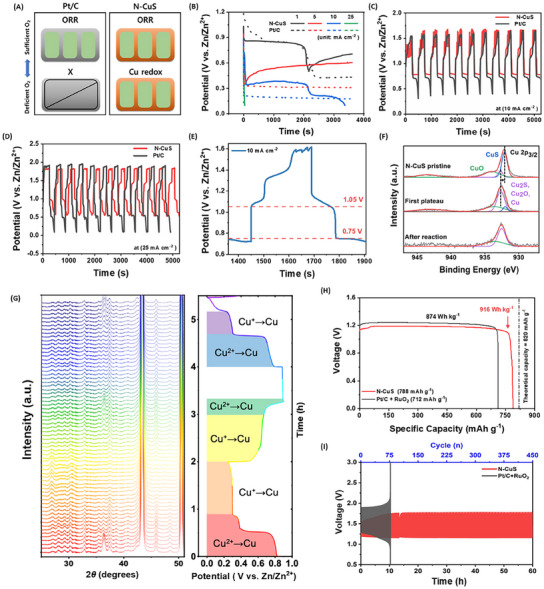
Electrochemical characterizations of a Zn–air battery (ZAB) with the N‐CuS cathode. A) Schematics of dual‐mode operations. B) Cell potentials under anaerobic conditions at the discharging rates of 1, 5, 10, and 25 mA cm^−2^. GCPL for 11 cycles at the current density of C) 10 mA cm^−2^ and D) 25 mA cm^−2^ E) Redox potentials during a single charging/discharging cycle, with two plateaus observed during discharging at 1.05 V (CuO‐to‐Cu_2_O transition) and 0.75 V (Cu_2_O‐to‐Cu transition), and F) XPS spectra corresponding to CuO‐to‐Cu_2_O and Cu_2_O‐to‐Cu transitions. G) In situ XRD pattern of N‐CuS during the first and half cycles under anaerobic conditions. H) Specific capacities and I) cycle stability of the ZABs with N‐CuS and Pt+RuO_2_, measured in the ZAB‐cell configurations.

Assembling the N‐CuS cathode and Zn metal anode in a ZAB configuration demonstrates its capability to operate in oxygen‐free, anaerobic conditions. **Figure** **5**A highlights that the N‐CuS cathode leverages Cu redox reactions even in the absence of oxygen, in stark contrast to the leading ORR catalyst, Pt/C, which requires oxygen for operation. Chronopotentiometry analysis at various discharging rates, as revealed in Figure 5B, further confirms cell functionality. Additionally, GCPL analyses were conducted to evaluate performance changes across repeated charging and discharging cycles (Figure 5C,D). The N‐CuS cathode exhibited distinct potentials during charging/discharging cycles, with more pronounced verification at a current density of 25 mA cm^−2^ compared to 10 mA cm^−2^. Furthermore, we compared the Pt/C on different substrates, including Cu foam and carbon paper (Figure S14, Supporting Information), to estimate the effect of the substrate. Here we observed more stable cyclability when Pt/C was coated on Cu foam. N‐CuS demonstrated prolonged stability, proving that the N‐CuS can effectively operate in oxygen‐free conditions. The analysis of Cu redox reactions during discharging revealed two plateaus at 1.05 and 0.75 V (Figure 5E), corresponding to the reduction of Cu^2^⁺ in CuO through Cu⁺ in Cu₂O to Cu⁰ in metallic Cu. This cascade battery‐mode operation, characterized by copper oxide transitions (CuO → Cu₂O → Cu) during discharging, facilitated the generation of O₂ molecules necessary to sustain ORR under anaerobic conditions. These findings demonstrate that the N‐CuS cathode ensures stable operation even under oxygen‐deficient conditions. This capability overcomes the limitations of the Pt/C electrode, which exhibits severely restricted or no performance in the absence of oxygen. In addition, XPS analysis conducted after each stage of Cu reduction confirmed the presence of increased reduced copper species, as displayed in Figure 5F. The Cu 2p XPS spectra were deconvoluted to distinguish the oxidation states of copper. Initially, the combined contribution of Cu^0^ and Cu^1+^ species stands at 6.74%. However, the contribution escalates to 49.17% after the first plateau and further to 77.89% after the second plateau, indicating a significant rise in the number of reduced copper species under anaerobic conditions. Additionally, the lattice fringes of N‐CuS after cycling in anaerobic conditions, depicted in Figure S15 (Supporting Information), support the presence of reduced copper species.^[^ ^57^, ^58^ ^]^ Furthermore, phase transitions during Zn‐Cu mode operation were scrutinized using in situ XRD, as depicted in Figures 5G and S16 (Supporting Information). Despite the in situ coin cell experiencing a significant ohmic drop in its voltage due to its construction, its charging/discharging profile matched those of copper sulfide materials.^[^ ^59^ ^]^ The peaks at 2θ = 14°, 17°, 18°, and 21° are attributed to the polypropylene (PP) separator,^[^ ^60^ ^]^ while the largest peaks at 43.3° and 50.3° originated from the Cu (111) and (200) phases of the Cu foam substrate. Additional peaks between 2θ = 26 to 31° correspond to CuS^[^ ^42^, ^43^ ^]^ and Cu_2_S phases,^[^ ^39^, ^40^ ^]^ whereas Cu_2_O (111)^[^ ^41^ ^]^ at 2θ = 36.4° and the Cu_2_S (102)^[^ ^61^ ^]^ at 2θ = 37.4° initially showed high intensity but diminished during discharging. Peaks for Cu₂S (220)^[^ ^62^ ^]^ at 2θ = 46° and CuO (‐202)^[^ ^41^ ^]^ at 2θ = 49° exhibited similar behavior, as did the Cu(OH)₂ (002)^[^ ^63^ ^]^ at 2θ = 33°. These observations suggest that CuO is first reduced to Cu₂O during discharging, followed by Cu_2_O's reduction to Cu, and vice versa during charging. In addition, electrochemical performance was evaluated in a ZAB cell with a zinc foil anode (Figure 5H,I). The N‐CuS cell delivered a specific capacity of 788 mAh g⁻¹ (Figure 5H), corresponding to 96% of the theoretical specific capacity (820 mAh g⁻¹), as well as a high gravimetric energy density of 916.0 Wh kg⁻¹, comparable to the theoretical energy density (1084 Wh kg⁻¹).^[^ ^64^ ^]^ Notably, these ZAB capacity and energy density outperform the state‐of‐the‐art Pt/C + RuO₂ cell (712.43 mAh g⁻¹ and 874 Wh kg⁻¹). Under Ar purging, the N‐CuS cathode exhibited a specific capacity of 798.8 mAh g⁻¹ (Figure S17A, Supporting information). The overall performance of the N‐CuS‐based ZAB shows superior capacity and gravimetric energy density compared to previously reported transition metal catalysts (Table S5, Supporting Information). Additionally, cycling stability tests, depicted in Figure 5I and Figure S17B (Supporting Information), demonstrate that a ZAB with the N‐CuS cathode enables stable operation sustaining over 450 cycles, resulting in approximately threefold longer cyclability than a ZAB with the Pt/C+IrO_2_ cathode, and sixfold longer cyclability than one with the Pt/C+RuO_2_ cathode. To further validate the high stability and low potential gap between charge and discharge, the electrochemical impedance spectroscopy (EIS) analysis of the ZAB was conducted, as illustrated in Figure S18A (Supporting Information). The obtained data were then fitted to the equivalent circuit model depicted in Figure S18B (Supporting Information). R_s_, R_int_, and R_ct_ represent the solution resistance, interfacial resistance, and charge transfer resistance, respectively. N‐CuS exhibited R_s_, R_int_, and R_ct_ values of 3.64, 0.86, and 3.25 Ω, while Pt/C+RuO₂ exhibited higher values of 5.28, 10.68, and 17.07 Ω. The substantially lower R_int_ and R_ct_ for N‐CuS indicate highly active interfacial characteristics at the electrode/electrolyte interface and enhanced ion accessibility. After the cycling test, the oxidized copper structure was observed (Figure S19, Supporting Information). The structural information obtained from the XRD pattern was consistent with the lattice pattern gained from the TEM measurement, without any noticeable degradation, suggesting the good stability of N‐CuS (Figure S20, Supporting Information).

**Figure 6:**
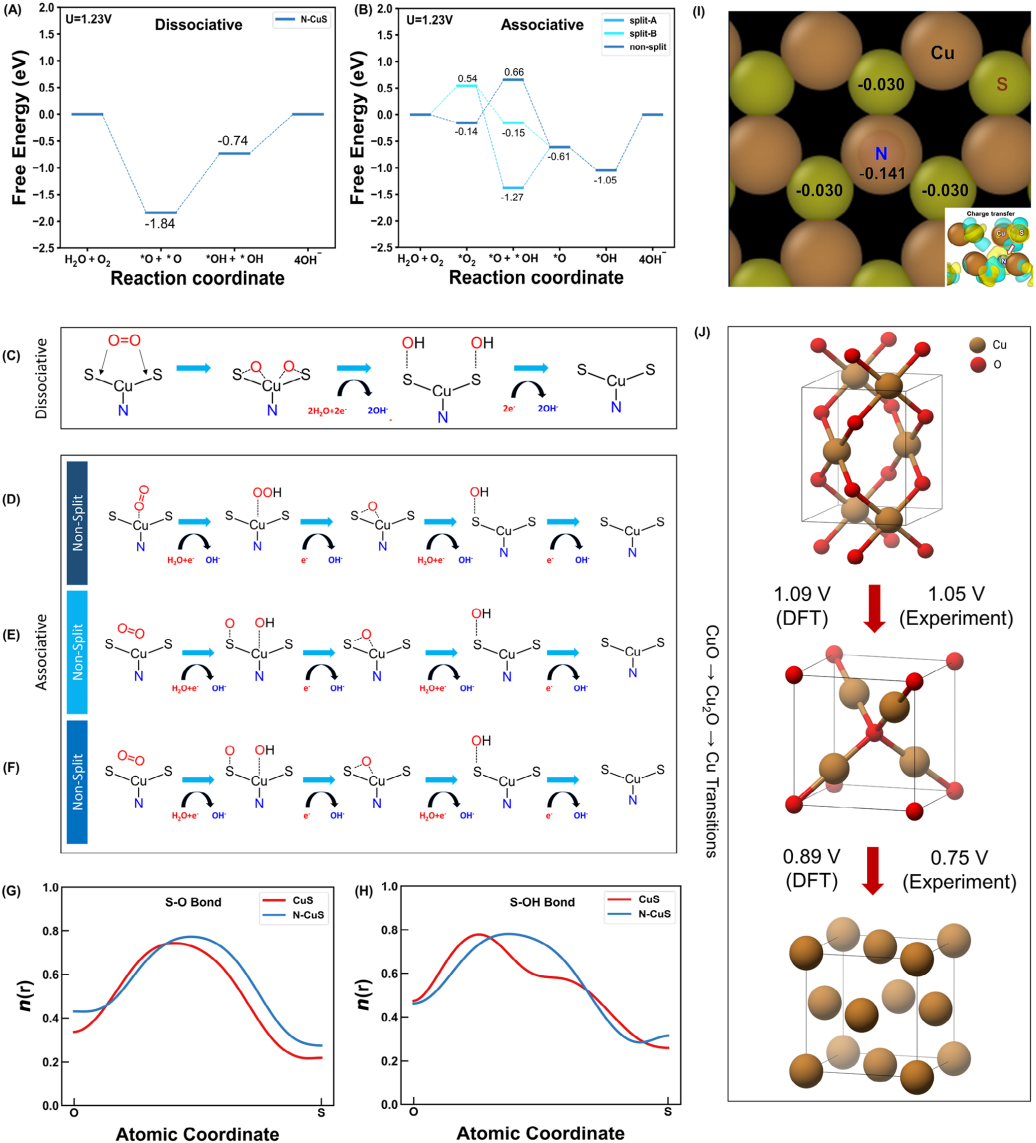
ORR and redox mechanisms from DFT calculations in aerobic and anaerobic conditions. Free energy diagrams for A) dissociative and B) associative ORR mechanisms on the N‐CuS surface at U = 1.23 V. Pathways for C) dissociative, D) non‐split associative, E) split‐A associative, F) split‐B associative ORR mechanisms on the N‐CuS surface. The electron localization function, *n*(r), was calculated along the line passing through G) S─O or H) S─OH bond. I) Bader charge and charge density difference (Δρ = ρ_N‐CuS_ – ρ_CuS_ – ρ_N_) plots for Cu and S atoms after N doping. The yellow regions represent electron accumulation, while the sky blue regions indicate electron depletion, with an isosurface level of 0.01e Å^−3^. J) Copper oxide transitions during discharging in the N‐CuS‐based ZAB under anaerobic conditions. The overpotentials from both experimental results and DFT calculations are provided.

The mechanisms governing ORR on CuS and N‐CuS were explored using the thermodynamic free energy diagrams derived from DFT calculations. **Figure** **6**A,B illustrates the Gibbs free energy diagrams for dissociative and associative pathways, respectively. On the N‐interstitial doped surface, one dissociative pathway (Figure 6C) and three different associative pathways – non‐split associative (Figure 6D), split associate A (Figure 6E), and split associate B (Figure 6F) – were identified. The theoretical overpotentials were calculated as 0.7 V on CuS, and 0.55, 1.05, 1.05, and 1.05 V for the dissociative, non‐split associative, split associative A, and split associative B pathways, respectively, on N‐CuS. These results indicate lower theoretical overpotentials for all pathways on N‐CuS compared to pristine CuS. The associative pathway initiates with O_2_ chemisorption on the electrode surface (*O_2_) on both N‐CuS and CuS, as depicted in Figure 6D–F and Figure S21 (Supporting Information), respectively. On the other hand, Figure S22 (Supporting Information) indicates that O_2_ does not adsorb in either side‐on or end‐on orientations on CuS. Figure S23A–D (Supporting Information) further illustrates that the spontaneous dissociation of the O═O bond occurs as each of the O atoms approaches S on the surface, supporting the predominance of the dissociative mechanism for ORR on the CuS surface. Similarly, the ORR mechanism on the N‐CuS surface was explored. DFT calculations, presented in Figure S23E–H (Supporting Information), reveal that the O═O bond cleaves more efficiently on N‐CuS compared to CuS. This finding is corroborated by the free energies before and after O_2_ bond dissociation at both surfaces, with N‐CuS favored by 0.97 eV for O_2_ bond dissociation. Following O_2_ adsorption in the dissociative pathway, intermediate O atoms are protonated to form *OH adsorbates, as depicted in Figures S24 and S25 (Supporting Information). On the N‐CuS surface, O_2_ can adsorb in two distinct configurations: side‐on at Cu─S or end‐on at Cu (Figure S26, Supporting Information). This demonstrates the important role of nitrogen doping in both associative and dissociative ORR pathways. In the end‐on orientation, ORR proceeds through an associative pathway (Figure S27, Supporting Information), with the adsorption site transitioning from Cu to a Cu─S bridge site after OH^‐^ desorption from *OOH. This mechanism is termed a non‐split associative pathway. In the side‐on configuration, the subsequent ORR route is determined by a protonation location on the oxygen. Further H^+^ adsorption on both O atoms leads to configurations A and B at the *O+*OH + H_2_O_(l)_ + OH^‐^ + 3e^‐^ step. In configuration A, H^+^ adsorbs on the O above the S site, while the O over the Cu atom relocates to the Cu─S bridge site. In configuration B, H^+^ adsorbs on the O above the Cu atom, followed by a relocation to the S site. The O on S undergoes protonation and desorbs as OH^‐^_(aq)_. These pathways are referred to as split‐associative pathways A and B, respectively. To further elucidate the effect of N doping on ORR activity, electron localization function (ELF)^[^ ^65^ ^]^ analysis was conducted to characterize S‐O_ads_ and S‐OH_ads_ chemical bonds, revealing the increased covalency of S─O bonding with intermediates *O and *OH on the surface (Figure 6G,H). Additionally, Bader charge analysis^[^ ^66^ ^]^ and charge density difference plots demonstrate that the doped N atom attracts electrons from its surroundings (Figure 6I), reducing electron density on adjacent Cu and S atoms by 0.141 and 0.030, respectively. Furthermore, in oxygen‐free conditions, copper oxide transitions for CuO → Cu_2_O and Cu_2_O → Cu occurred during discharging. DFT calculations (Figure 6J) reveal the redox voltages of 1.09 V for CuO → Cu_2_O and 0.89 V for Cu_2_O → Cu, agreeing with the experimental voltages of 1.05 and 0.75 V, respectively. Table S6 (Supporting Information) summarizes the details of DFT functionals and calculation parameters. The redox cell voltages were determined by using Gibbs free energies and the Nernst Equation^[^ ^67^ ^]^ at pH 14 for both anode and cathode reactions. These findings demonstrate the stability of the N‐CuS cathode under oxygen‐deficient conditions.

## 3 Conclusion

In summary, we synthesized the N‐CuS cathode and demonstrated its efficacy as a bifunctional OER/ORR catalyst, delivering high performance in ZABs. The anodizing chemical process was used to generate abundant redox‐active surfaces where the electrolyte facilitated copper sulfide formation. N dopants were then incorporated into the copper sulfide catalyst to result in N‐CuS with conductive N 2p – S 3p hybridized orbitals, oxygen‐transporting mesopores, and a high electrochemically active surface area. Moreover, N‐CuS exhibited dual‐functionality in cathodic reactions. The battery operated in a ZAB mode under aerobic conditions by converting ambient oxygen into hydroxides, while residual copper oxides operated in a Zn‐Cu mode under anaerobic conditions by serving as redox sites. The ZAB assembled with N‐CuS cathode and Zn metal anode electrodes achieved a high capacity of 788 mAh g^−1^, corresponding to 96% of the theoretical capacity. Also, the high discharging cell voltage resulted in a remarkably high gravimetric energy density of 916.0 Wh kg^−1^ under aerobic conditions. DFT calculations further elucidated that O_2_ dissociation is 0.97 eV more favorable on N‐CuS than on CuS. Surface adsorbed O atoms are subsequently protonated to form *OH adsorbates via a dissociate pathway, exhibiting an overpotential of 0.55 V, outperforming bare CuS at 0.7 V. Alternatively, in the presence of adjacent sulfur, H^+^ adsorbs on surface‐adsorbed O atoms to form *OH through non‐spit associate, split associate A, or split associate B pathway with an overpotential of 1.05 V. Then, *OH desorbs from Cu in an aqueous solution as OH^‐^_aq_. These findings revealed that nitrogen doping not only enhances the dissociative ORR mechanism but also facilitates the associative ORR pathway, demonstrating the important role of nitrogen doping. However, under anaerobic conditions, copper oxides in N‐CuS underwent reduction via phase transitions (CuO →Cu_2_O→Cu) with the discharging voltages of 1.05 and 0.75 V, resulting in the formation of oxygen to sustain ORR. Conversely, during charging, the reverse transitions (Cu→Cu_2_O→CuO) occurred via oxidation by oxygen produced through OER. These cascade transitions enabled a ZAB with the N‐CuS cathode to operate without power loss, even when the air supply is interrupted, supporting that the N‐CuS electrode ensures stable performance under oxygen‐deficient conditions. Additionally, a ZAB with the N‐CuS cathode achieved approximately threefold longer cycle life compared to a ZAB with the Pt/C+IrO_2_ cathode or about sixfold longer cycle life than a ZAB with the Pt/C+RuO_2_ cathode. Consequently, we believe that this work paves the way for realizing efficient ORR/OER via dual‐mode cathode reactions, providing high energy density and cycle stability in ZABs for advanced applications across various fields.

## 4 Experimental Section

### Materials

Copper foam (0.5 mm, MTI Korea), Na_2_S∙9H_2_O (Sigma Aldrich, ACS reagent, ≥98.0%), KOH (Sigma–Aldrich, ACS reagent, ≥85%), HClO_4_ (Sigma–Aldrich, ACS reagent, 70%), Zn foil (0.25 mm, Thermo Fisher Scientific), Zn foil (0.05 mm, MTI Korea), gas purging cell (gas diffusion flow cell, WizMAC), zinc–air battery (ZAB) cell (OMS‐T1, Spring New Energy), GDL (PTFE‐coated carbon sheet, Sigracet, 39BB), carbon sheet (TGP‐H‐120, Toray), anion exchange membrane (Nafion‐115 membrane, Alfa Aesar, 0.125 mm, 0.9 mEq g^−1^).

### Synthesis of Copper Sulfide (CuS)

Copper sulfide nanoflakes were prepared by the electrochemical anodization method. The Cu foam was washed with acetone and distilled water. The cleaned Cu foams were used as an anode and the carbon paper was a cathode with 1.5 m Na_2_S electrolyte solution. Then an oxidation current of 10 mA was discharged for 10 min at 0 °C. The obtained samples were washed with distilled water several times and dried at room temperature.

### Preparation of Nitrogen‐Doped Copper Sulfide (N‐CuS)

N_2_ plasma treatment was carried out with plasma‐enhanced chemical vapor deposition (PECVD, Woosin CryoVac, Korea). To prevent the nanoflakes from being destructed due to direct exposure, a Si shield is added on top of the copper sulfide nanoflakes.

### Preparation of PGM Catalyst Ink

The amount of the catalyst material cast on the copper foam substrate has been assessed using the inductively coupled plasma optical emission spectroscopy (ICP‐OES, Agilent) and XPS analysis in conjunction (Tables S4 and S7, Supporting Information). From the ICP‐OES and deconvoluted XPS oxidation state ratio analyses, the catalyst material loading density on the Cu foam substrate was determined to be 1.229 mg cm^−2^. In case of Pt/C electrodes, catalyst ink was prepared by suspending 10 mg of Pt/C (20%wt Pt, Sigma–Aldrich) in a mixture of 900 µL isopropyl alcohol and 100 µL Nafion solution (5% Nafion 1100 W solution, Sigma–Aldrich) by sonication for at least 15 min. In case of Pt/C + RuO_2_ electrodes, catalyst ink was prepared by suspending 5 mg of Pt/C and RuO_2_ (Sigma–Aldrich) each, in a mixture of 900 µL isopropyl alcohol and 100 µL Nafion solution by sonication for at least 15 min.

### Preparation of Pt/C onto Cu Foam and Carbon Paper Control Electrode

The catalyst ink prepared as described above was drop‐cast onto carbon paper and copper foam substrates. Each sample was coated with 123 µL of the abovementioned ink. The ink was dropped at a maximum volume of 25 µL per drop, and subsequent drops were added after the previous one was fully dry.

### Half‐Cell Measurement Configuration

A three‐electrode half‐cell measurement was conducted using an H‐type cell equipped with a membrane separator to evaluate oxygen reduction/evolution reaction (ORR/OER) performance. A Pt coil served as the counter electrode, while a Hg/HgO electrode was used as the reference electrode. About 1 m KOH was used as the electrolyte. A Nafion‐115 membrane with a thickness of 0.125 mm and an exchange capacity of 0.9 mEq g^−1^ was employed as the separator.

### Gas Purging Cell Configuration (Ar or O_2_ Gas)

A gas purging cell separated with a membrane was adopted for Zn–air cell electrochemical measurements. First of all, Zn metal was prepared as the anode. The N‐CuS and carbon paper with a mesoporous layer were used for the cathode electrode. Anode and cathode electrodes were attached with copper tape. About 6 m KOH electrolyte and anion exchange membrane (Nafion 115 membrane, as mentioned above) were applied to the electrolyte and separator, respectively.

### Zinc–Air Battery (ZAB) Configuration (Ambient Air)

A Zn sheet (0.25 mm) was polished and washed before being used as the anode. The N‐CuS, used as the air cathode, was cut to fit the gas inlet of the ZAB cell and covered with a GDL to prevent leakage. A Ni foam was assembled as a backing layer next to the GDL for both the current collector and support for the GDL. A 6 m KOH + 0.2 m Zn(OAc)_2_ solution was selected as the electrolyte. The specific capacity and energy density of the ZAB were calculated based on the mass of the consumed Zn sheet after discharging at a current density of 10 mA cm^−2^.

### In Situ X‐Ray Diffraction (XRD)

A coin cell using N‐CuS, hole‐bored zinc disc (14 mm in diameter, 0.50 mm in thickness, 3 mm hole diameter), and a PP cell separator were assembled. The cell casings had ≈3 mm of hole bored through, sealed with mylar film and epoxy. In situ XRD spectra were observed (XRD, Rigaku R‐Axis IV) while cycling at a charging/discharging rate of 0.5 mA cm^−2^.

### Characterization

The images of CuS and N‐CuS for the morphological study were captured using the field emission scanning electron microscope (FESEM, Hitachi, SU8230 and FEI company, Magellan 400) and high‐resolution transmission electron microscope (Thermo Fisher, Spectra Ultra, and JEOL, JEM‐ARM200F) image was taken of pulverized N‐CuS sample. The crystal structure analysis was performed by X‐ray diffraction (XRD, Rigaku SmartLab) using Cu Kα radiation operating conditions of 40 kV and 30 mA. To study surface chemical states, X‐ray photoelectron spectroscopy (XPS) and Auger electron spectroscopy (AES) analyses were performed by Kratos Axis‐Supra with Al (1486.7 eV) as the X‐ray source. X‐ray absorption spectroscopy (XAS – XANES/EXAFS) measurements were performed by the X‐ray open lab, advanced analysis center of KIST at Pohang Light Source (PLS‐II), KIST‐PAL.

### Electrochemical Measurement

Linear sweep voltammetry (LSV) was conducted from open circuit potential to 0.3 V versus RHE for ORR and from open circuit potential to 1.75 V versus RHE for OER under Ar purging and O_2_ purging conditions, respectively. Cyclic voltammetry (CV) was performed within a potential range of 0.3–1.2 V versus RHE to evaluate the reversible catalytic activity. The electrochemically active surface area (ESCA) was analyzed through multiple CV measurements conducted at various scan rates of 20, 40, 60, 80, and 100 mV s^−1^. ZAB performance was tested by Chronopotentiometry (CP) test in various discharging current density conditions, 1, 5, 10, 25 mA, and galvanostatic cycling with potential limitation (GCPL) at a current density of 10 mA cm^−2^, 25 mA cm^−2^ with the different gas condition, O_2_ and Ar purging, respectively. Cycle stability data were collected using GCPL at a charging/discharging rate of 1 mA cm^−2^, with potential limits set between 0.1 and 3.5 V. Each charging and discharging step lasted 4 minutes, resulting in a total cycle duration of 8 minutes. Half‐cell measurements, including ECSA analysis and OER/ORR assessments, were conducted with 100% iR compensation. However, all zinc battery measurements, whether performed in a gas‐purging cell or ZAB configuration, were carried out without iR compensation.

### Density Functional Theory (DFT) Calculations

The DFT calculations with a plane‐wave basis set were performed by Vienna ab initio simulation package (VASP)^[^ ^68^ ^]^ with the projector‐augmented‐wave (PAW) method.^[^ ^69^, ^70^ ^]^ The Perdew‐Burke‐Ernzerhof (PBE) functional^[^ ^71^ ^]^ with Hubbard U correction (U_eff_ = 5.0 eV)^[^ ^72^ ^]^ was implemented to deal with the exchange‐correlation part of the Hamiltonian within the generalized gradient approximation (GGA).^[^ ^73^ ^]^ All calculation was done in a spin‐polarized manner. For ORR‐related calculations, the effect of dispersive forces was taken into account using the DFT‐D4 method.^[^ ^74^ ^]^ The energy and force convergences were 10^−5^ eV and 0.04 eV Å^−1^, and the energy cut‐off of the plane wave basis set was 520 eV in all calculations. The Brillouin zone was sampled by a 6 × 6 × 1 k‐point grid for bulk optimization, 3 × 3 × 1 k‐point grid for the surface slab model and a vacuum space of more than 15 Å was guaranteed along the z‐direction to neglect the interaction between the subsequent slabs. In the context of anaerobic condition simulations for copper and its oxides (Cu, CuO, Cu_2_O), the Brillouin zone was sampled by a 7 × 7 × 7 k‐point grid for bulk optimization. The change in free energy at each step of ORR was determined using this formula: ΔG = ΔE + ΔZPE − TΔS. Here, ΔE, ΔZPE, and ΔS stand for the changes in enthalpy, zero‐point energy, and entropy, respectively. Calculations for free energy were done under the condition of the basic environment with a pH of 14. Even though the processes differ, the ORR in a basic environment still involves linked transfers of electrons and protons, just like in an acidic environment. According to Nørskov's method,^[^ ^75^ ^]^ the free energy of a pair of protons and electrons is the same as the free energy of 1/2H_2_ at an electrode potential of 0 V compared to the standard hydrogen electrode (SHE) at a pH of 0. When the pH is constant, the free energy of a system at a specific point in a reaction involving “n” number of electrons was adjusted by ‐neU where “U” represents the electrode potential value compared to the reversible hydrogen electrode (RHE). The number of linked transfers of electrons and protons during the ORR in a basic environment is essentially the same as in an acidic environment.

### Calculation Details for Redox Reactions Under Oxygen‐Free Conditions

Various methodologies have been utilized and investigated to identify the optimal functional and calculation parameters. Notably, the determination of an effective set of parameters significantly influences the outcomes of calculations for these materials. The initial magnetic moment on copper is a critical factor impacting the accuracy of calculations for bulk CuO. It is known that bulk CuO has an antiferromagnetic ground state,^[^ ^76^ ^]^ initial magnetic moment on copper greatly influenced the calculation results. Moreover, the inclusion of Van‐der‐Waals (VdW) correction in calculations significantly affects the results.

Table S6 (Supporting Information) shows the calculated standard potential for two cathode reduction reactions (CuO→Cu_2_O, Cu_2_O→Cu). Different VdW corrections, namely the DFT‐D3 method of Grimme with zero‐damping function,^[^ ^77^ ^]^ the DFT‐D3 method with Becke‐Johnson damping function,^[^ ^78^ ^]^ and the DFT‐D4 method were explored. Surprisingly, all dispersion corrections exhibited a reversed potential trend compared to experimental results. Consequently, subsequent redox reaction calculations were executed without VdW correction. Additionally, the choice of exchange‐correlation function significantly impacts the computational outcomes. Various functionals were considered, including Perdew–Burke–Ernzerhof (PBE),^[^ ^73^ ^]^ Revised PBE from Hammer et al. (RPBE)^[^ ^79^ ^]^ with Hubbard U correction (U_eff_ = 5.0 eV), Heyd‐Scuseria‐Ernzerhof functional (HSE06)^[^ ^80^ ^]^ with standard values of a = 1/4 and ω = 0.2, and regularized‐restored strongly constrained and appropriately normed (R2SCAN) functional.^[^ ^81^ ^]^ Notably, among the considered functionals, RPBE+U demonstrated the closest agreement with experimental potentials. Furthermore, the U_eff_ value of 4 eV exhibited results closest to the experimental data after U parameter fitting.

## Conflict of Interest

The authors declare no conflict of interest.

## Supporting information



Supporting Information

## Data Availability

The data that support the findings of this study are available from the corresponding author upon reasonable request.
