# Supplementary material for: Serrated Leaf‐Like N‐Doped Copper Sulfide Enabling Bifunctional Oxygen Reduction/Evolution via Dual‐Mode Cathode Reactions for High Energy Density and Cycle Stability in Zinc–Air Batteries
Source: Adv Sci (Weinh). 2025 Apr 4;12(20):2413425. doi: 10.1002/advs.202413425 (PMC12120742; doi:10.1002/advs.202413425)
Supplement: Supplementary file 1 — Supporting Information [file ADVS-12-2413425-s001.docx]

Copyright WILEY-VCH Verlag GmbH & Co. KGaA, 69469 Weinheim, Germany, 2025.

Supporting Information

Serrated Leaf-like N-Doped Copper Sulfide Enabling Bifunctional Oxygen Reduction/Evolution via Dual-Mode Cathode Reactions for High Energy Density and Cycle Stability in Zinc-Air Batteries

Do Hwan Jung^‡^, Yong Hak Park^‡^, Dong Won Kim^‡^, Jong Hui Choi^‡^, Seungrae Cho, Keon-Han Kim, Dong Gyu Park, Byungchan Han*, and Jeung Ku Kang*

D.H. Jung, D.W. Kim, Dr. J.H. Choi, S. Cho, Dr. D.G. Park, Prof. J.K. Kang

Department of Materials Science and Engineering and NanoCentury Institute

Korea Advanced Institute of Science and Technology (KAIST)

291 Daehak-ro, Yuseong-gu, Daejeon 34141, Republic of Korea

Y.H. Park, Prof. B.C. Han

Department of Chemical and Biomolecular Engineering

Yonsei University

50 Yonsei-ro, Seodaemun-gu, Seoul 03722, Republic of Korea

Dr. K-H. Kim

The Research Institute of Basic Science

Seoul National University

1 Gwanak-ro, Gwanak-gu, Seoul 08826, Republic of Korea

*^‡^*D.H. Jung, Y.H. Park, D.W. Kim, and Dr. J. H. Choi contributed equally to this work

^*^Corresponding authors: bchan@yonsei.ac.kr, jeungku@kaist.ac.kr

**Supporting figures and tables**


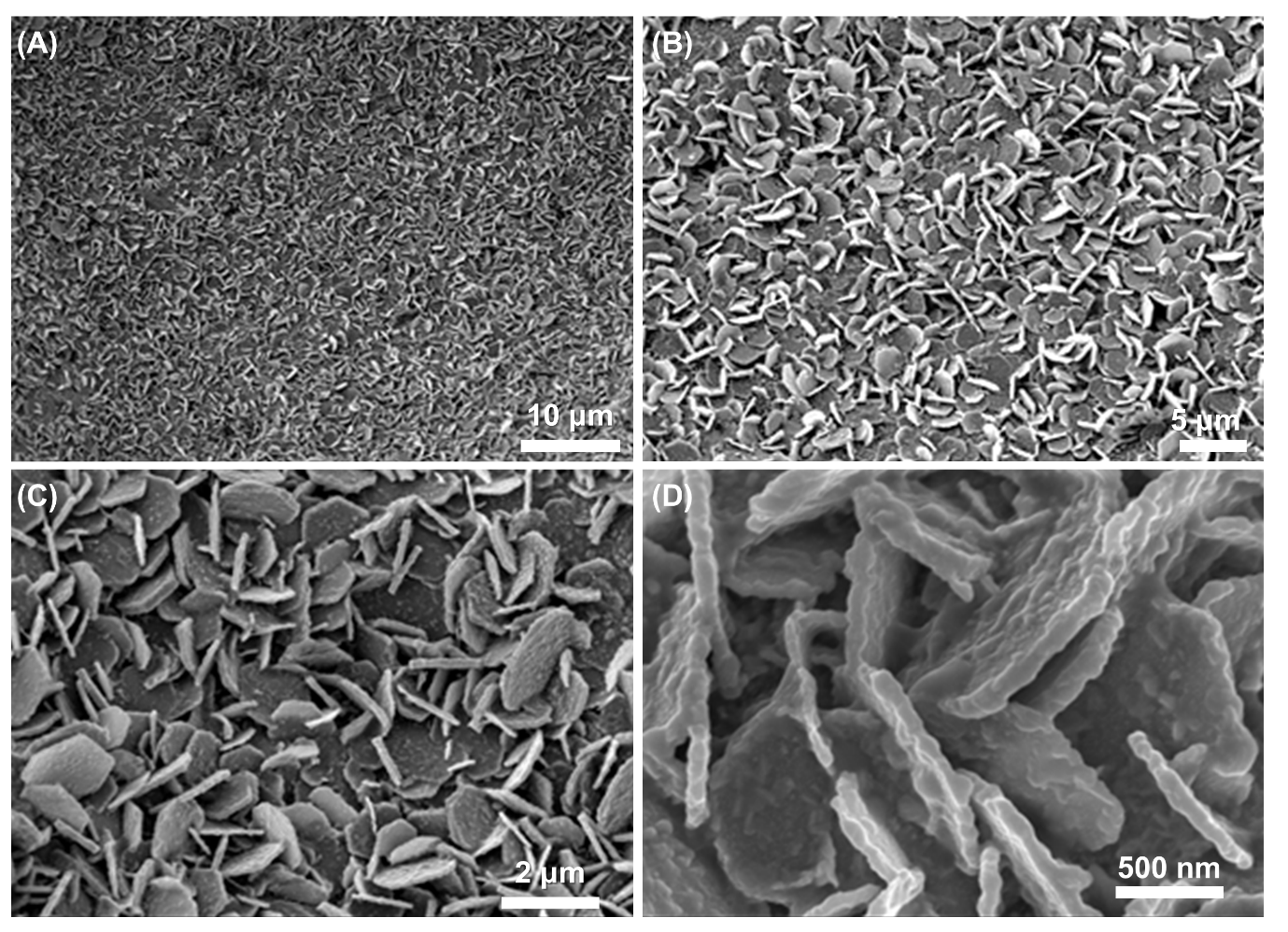


Figure S1. Scanning electron microscopy (SEM) images of CuS with different magnifications.


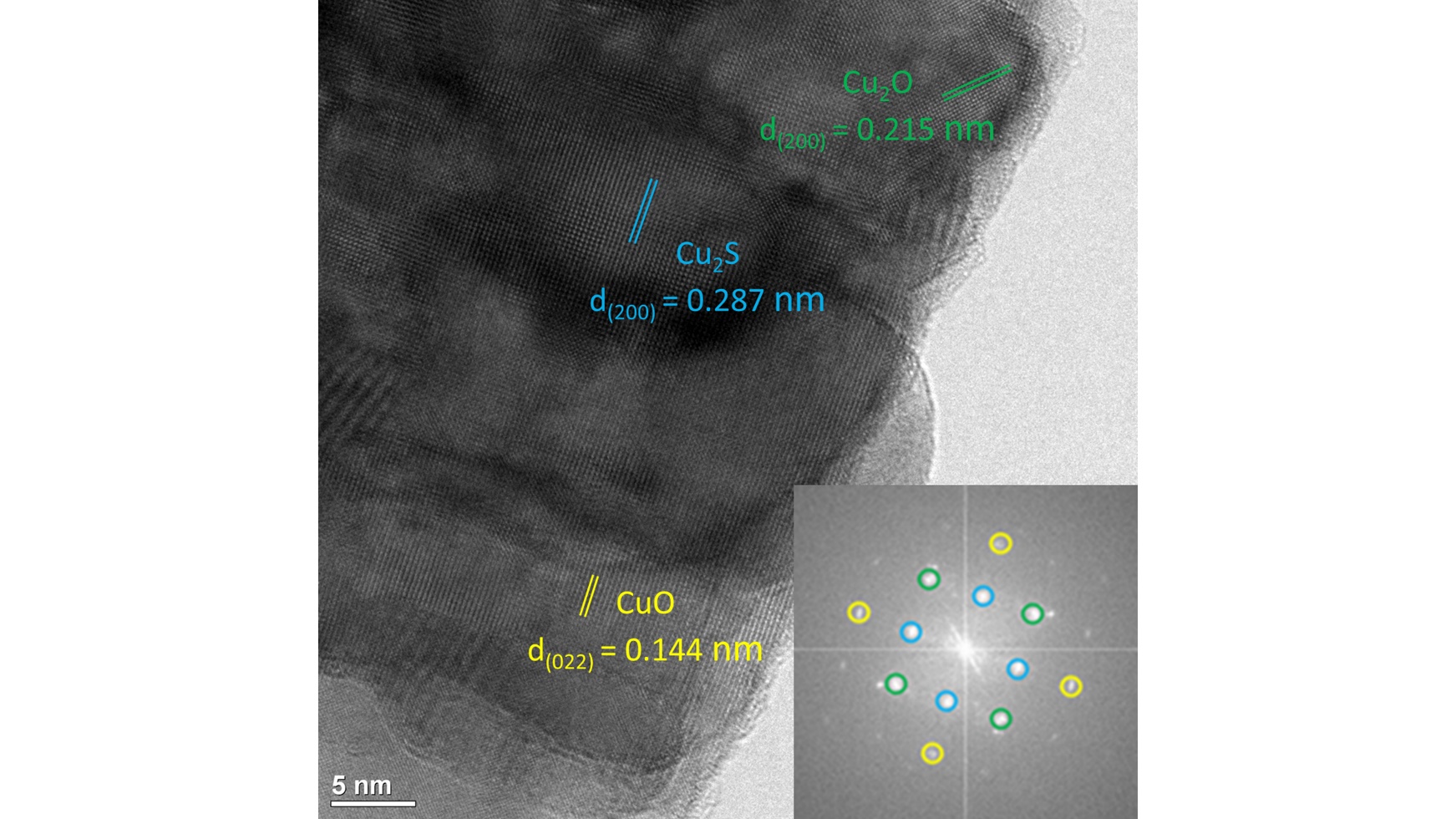


Figure S2. Transmission electron microscopy (TEM) image of N-CuS.


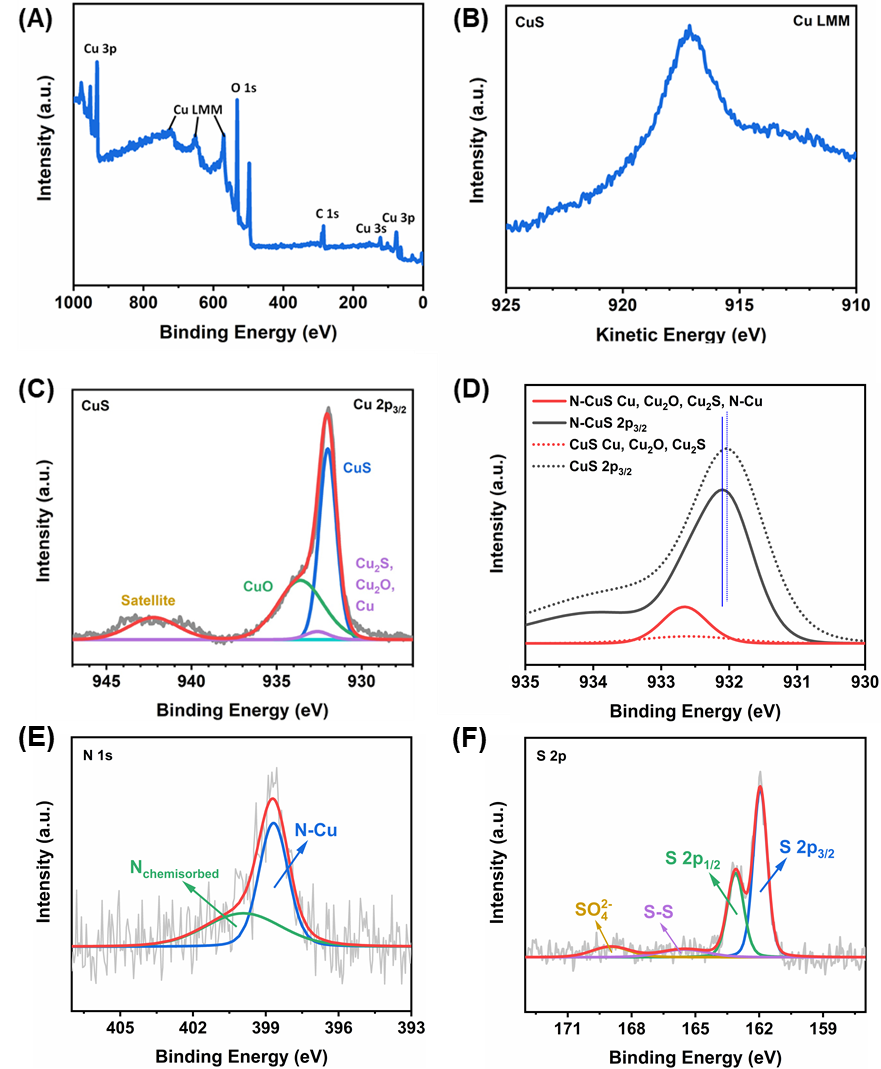


Figure S3. X-ray photoelectron spectroscopy (XPS) analyses of CuS and N-CuS. (A) Survey spectrum of CuS. (B) Cu LMM area spectrum of CuS. (C) Deconvoluted Cu 2p_3/2_ of CuS. (D) N_2_ Plasma treat induced spectra shift between CuS and N-CuS of Cu 2p_3/2_, where solid lines are N-CuS and dashed lines are CuS. (E) N 1s and (F) S 2p spectra of N-CuS.

**
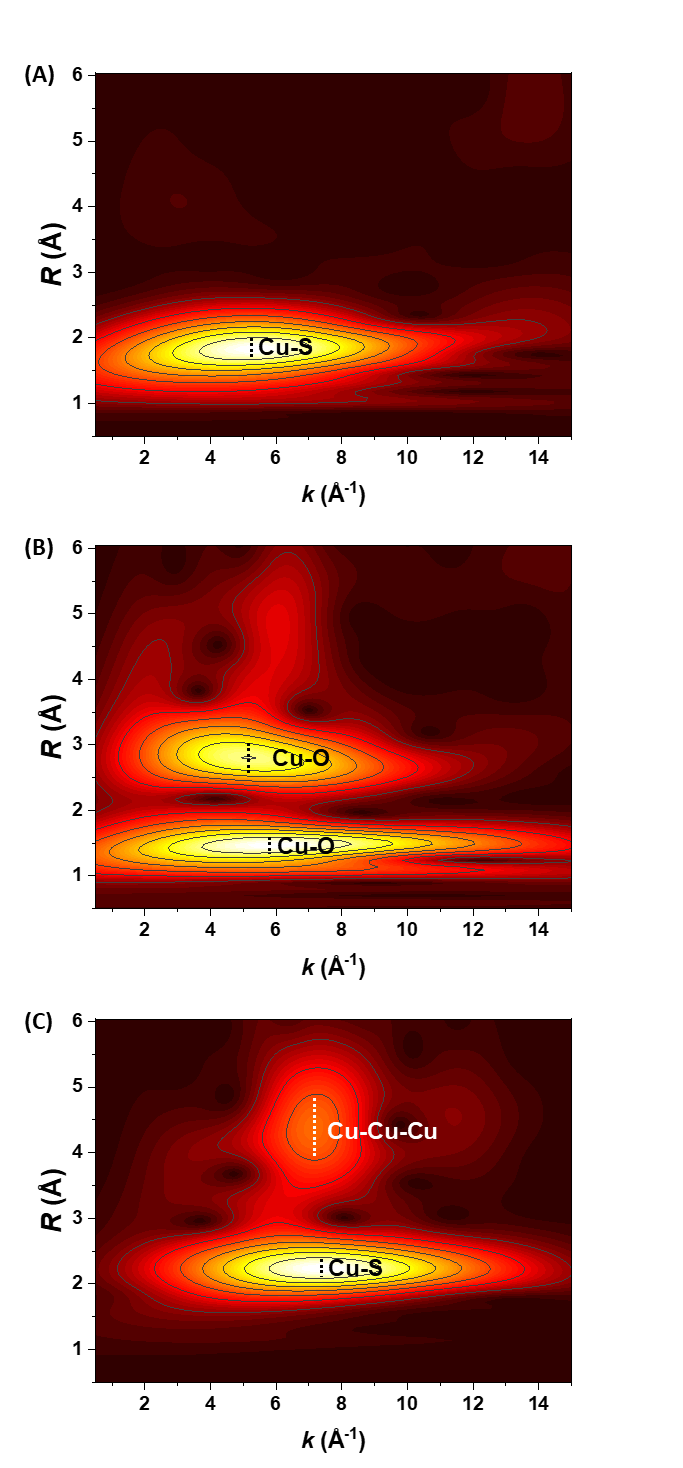
**

**Figure S4. Morlet wavelet transform (WT) plots.** Those for (A) Cu_2_S (B) Cu_2_O and (C) Copper foil.


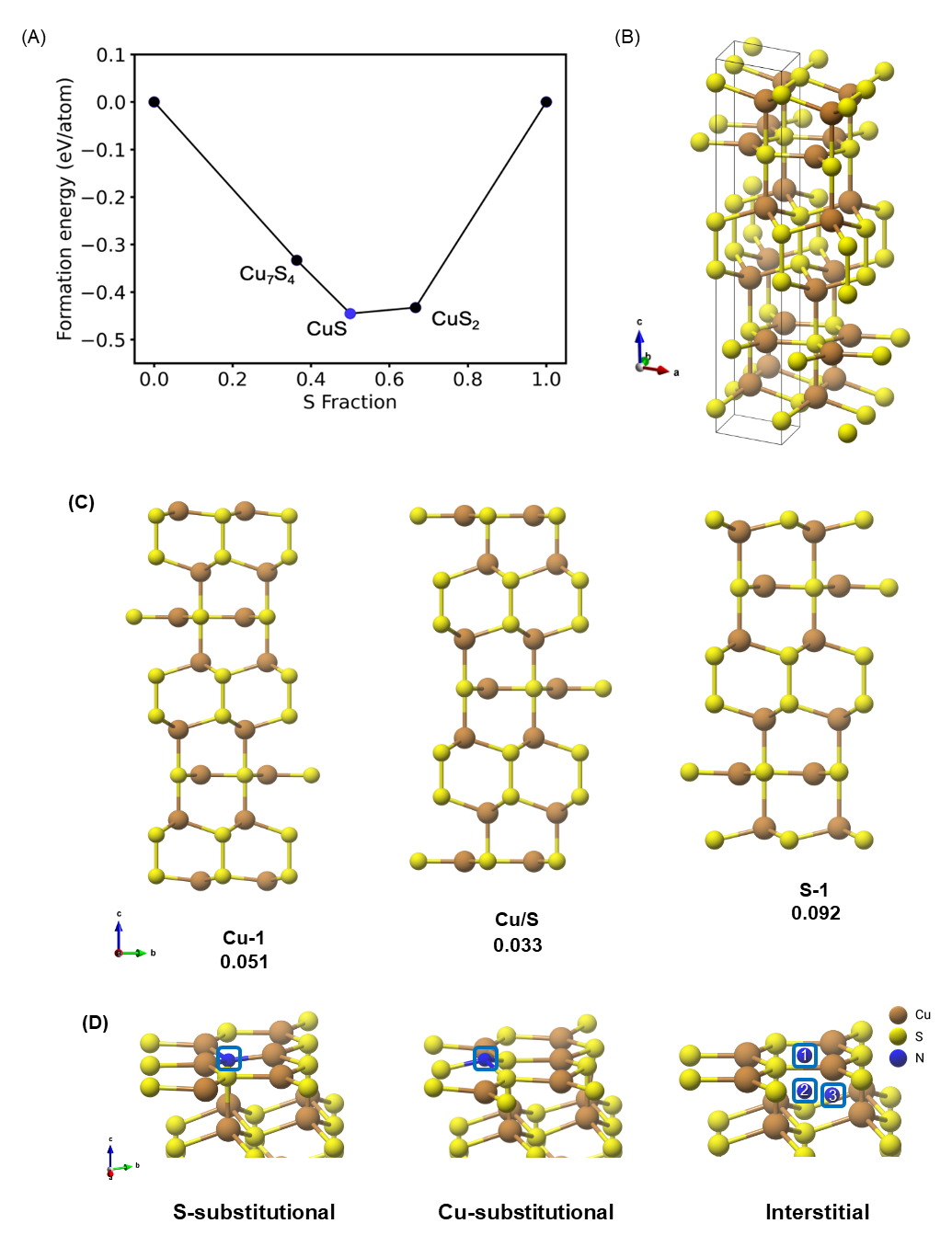


**Figure S5. Surface models for density functional theory (DFT) calculations**. (A) Energy convex hull of bulk copper sulfide formations as a function of S composition and the thermodynamically most stable Covellite structure was identified (blue circle) with the atomistic structure in (B). (C) Calculated surface energies (eV/Å2) of different terminations of the covellite CuS (001) surfaces. The name of each termination is indicated above the surface energy. (D) Considered N-doping configurations. The energies for substitutional and interstitial doping are detailed in Table S2.


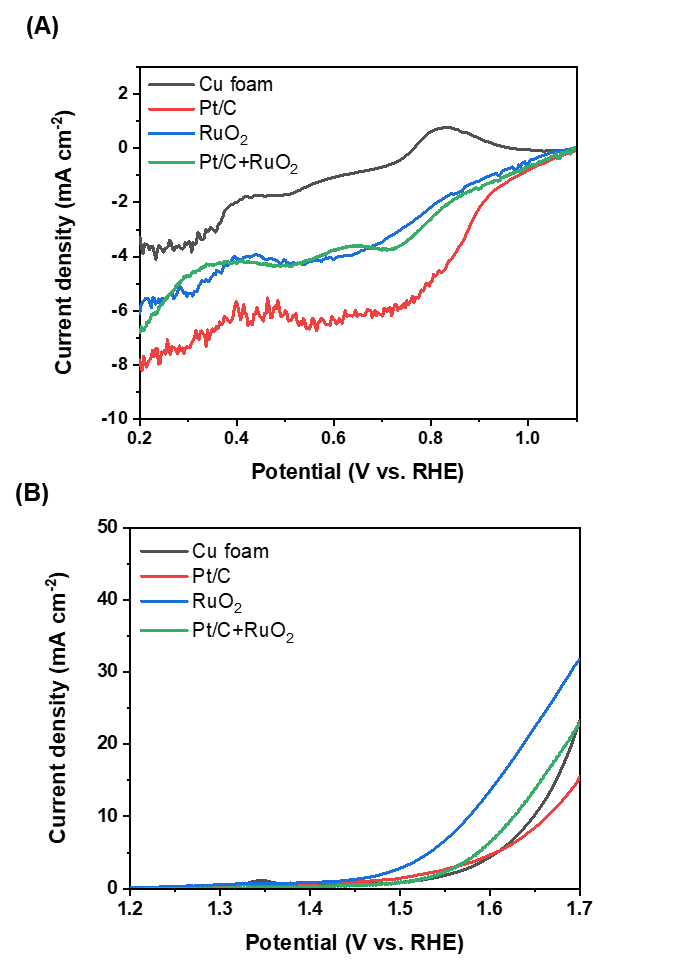


**Figure S6. Electrochemical performance of bare Cu foam, Pt/C, RuO_2_, and Pt/C+RuO_2_.** (A) Linear sweep voltammetry (LSV) curves for ORR and (B) LSV curves for OER. The Pt/C, RuO_2_, and Pt/C+RuO_2_ samples were prepared by drop casting onto Cu foam.

**
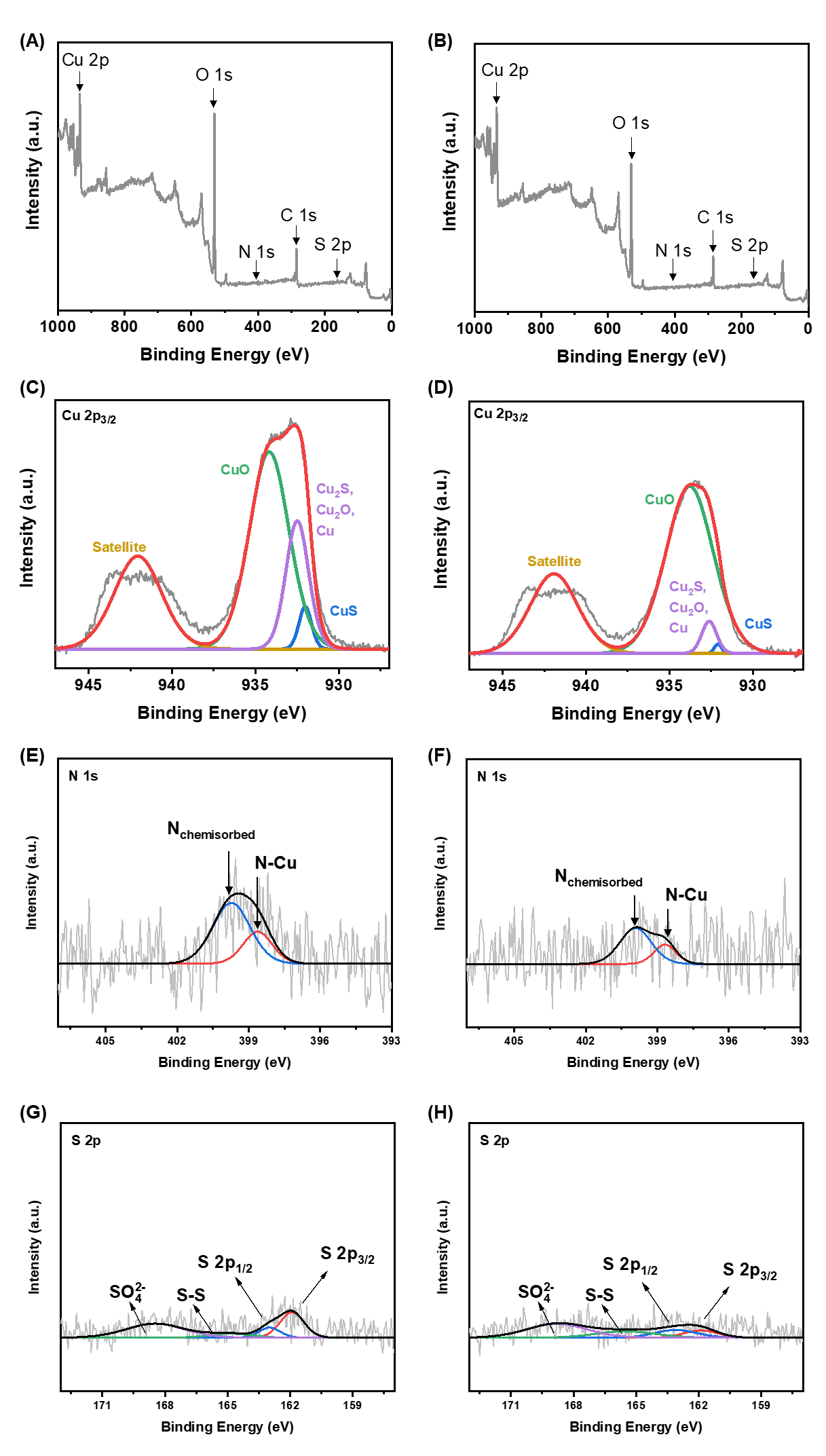
**

**Figure S7. *Ex-situ* XPS of N-CuS.** (A-B) survey scan, (C-D) Cu 2p_3/2_, (E-F) N 1s, and (G-H) S 2p. Spectra of A, C, E, and G were obtained after the ORR at 1.6 V vs. RHE, while spectra B, D, F, and H were obtained after the OER at 0.2 V vs. RHE.

**
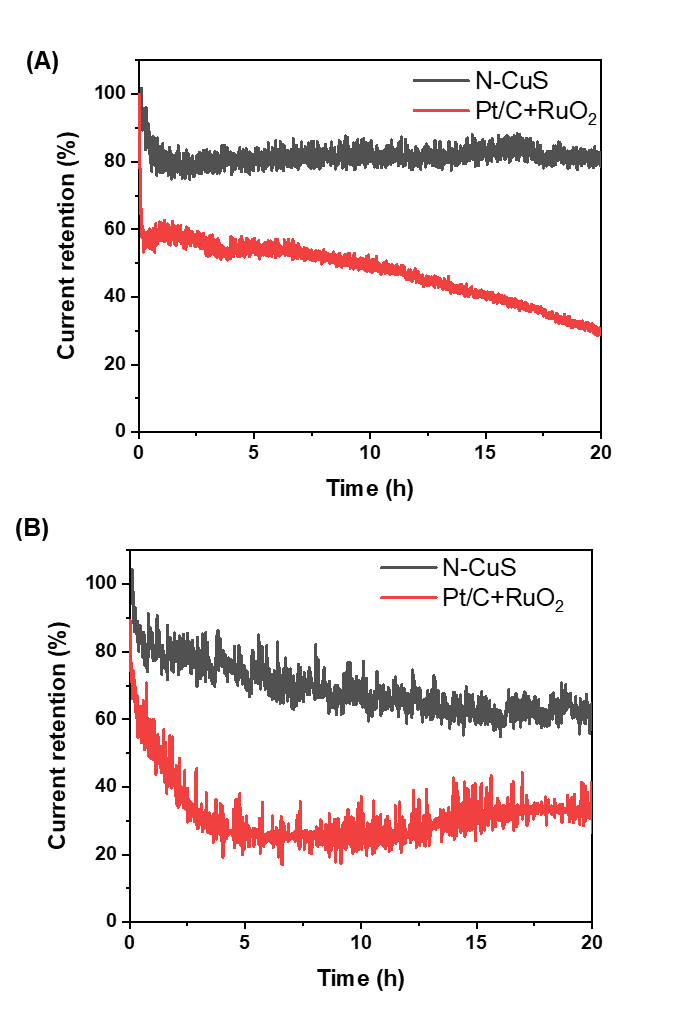
Figure S8.** Stability tests of N-CuS and commercial Pt/C+RuO_2_ on Cu foam. (A) ORR at 0.6 V vs. RHE and (B) OER at 1.6 V vs. RHE.


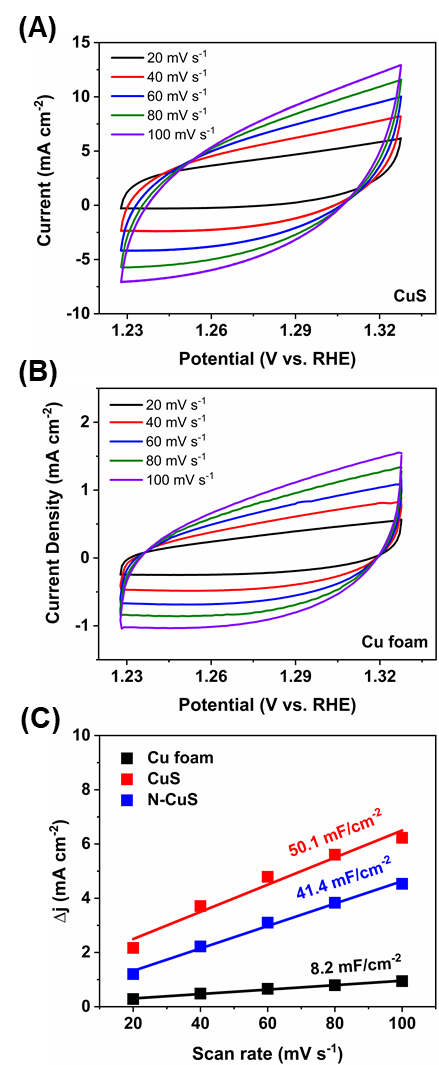


Figure S9. Cyclic voltammetry (CV) plots and calculated C_dl_ values. Those for (A) pristine CuS, (B) Cu metal foam with various scan rates of 20, 40, 60, 80, and 100 mV s^-1^ where 1M KOH solution was used as an electrolyte. (C) Average current density ($\boldsymbol{\Delta}$j=(j_a_-j_c_)/2) versus the scan rate plot and corresponding calculated C_dl_ values of three samples.

**
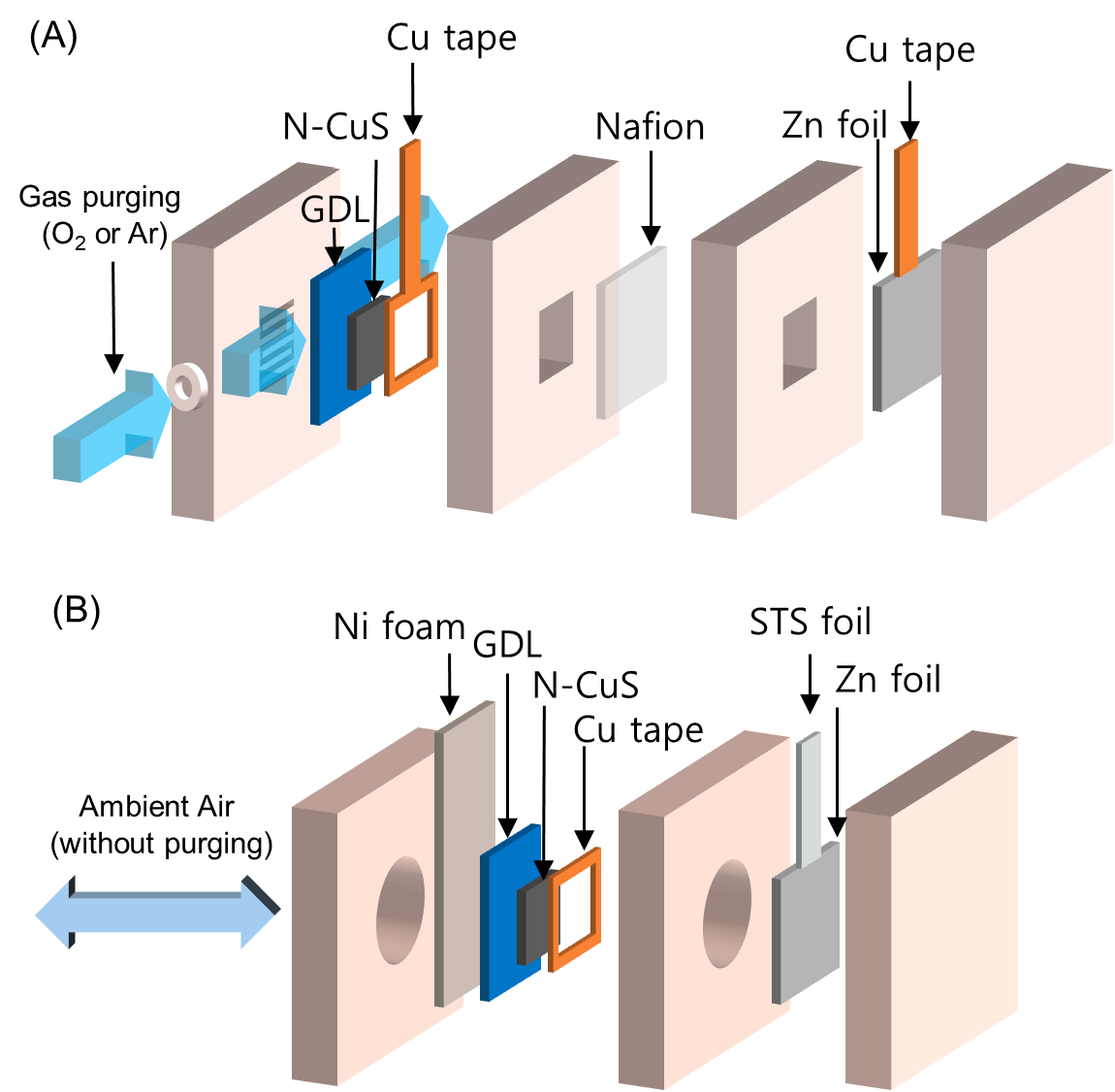
**

**Figure S10. Cell configurations for gas-purging experiments under aerobic and anaerobic conditions, along with the Zinc-air battery (ZAB) configuration, which operates using ambient air without purging.** (**A**) A cell with a continuous gas flow of O₂ or Ar, and (**B**) a ZAB functioning in ambient air conditions without an additional purging process.


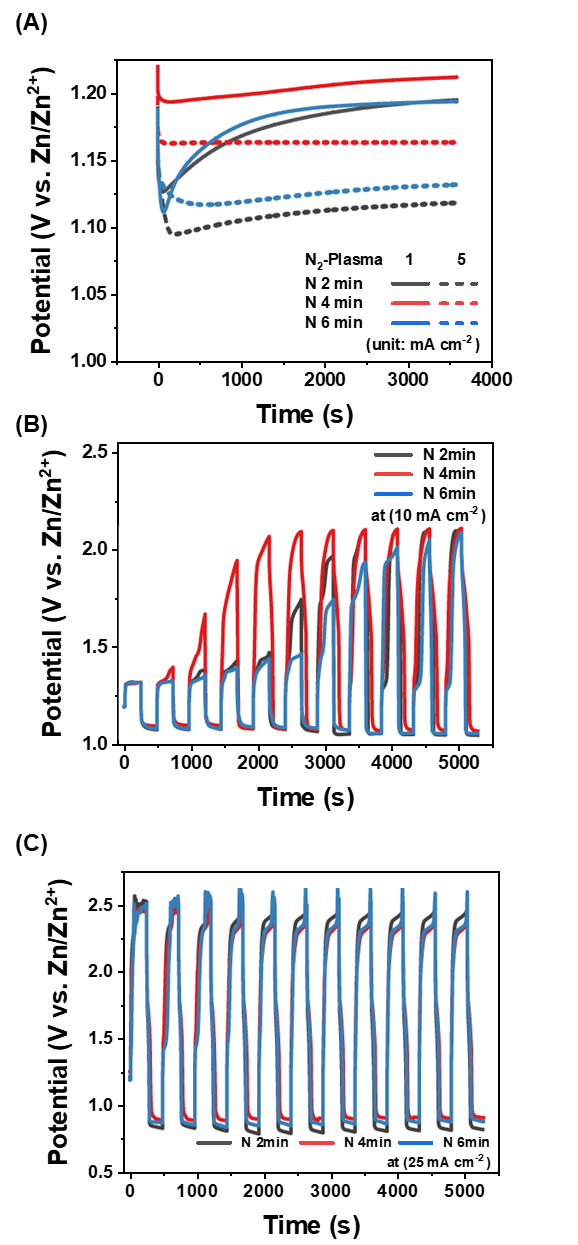


**Figure S11. Electrochemical performances of the N-CuS cathode on the varying N_2_-plasma treatment time, which is 2 min, 4 min, and 6 min in aerobic condition (O_2_ purging). (A)** Chronopotentiometry test curves measured at the current densities of 1 and 5 mA cm^-2^. Galvanostatic charge and discharge at **(B)** 10 mA cm^-2^ and **(C)** 25 mA cm^-2^.

**

**

**Figure S12. Discharge rate performance of N-CuS and Pt/C gas purging cell on Cu foam under aerobic conditions (O_2_ purging).**

**
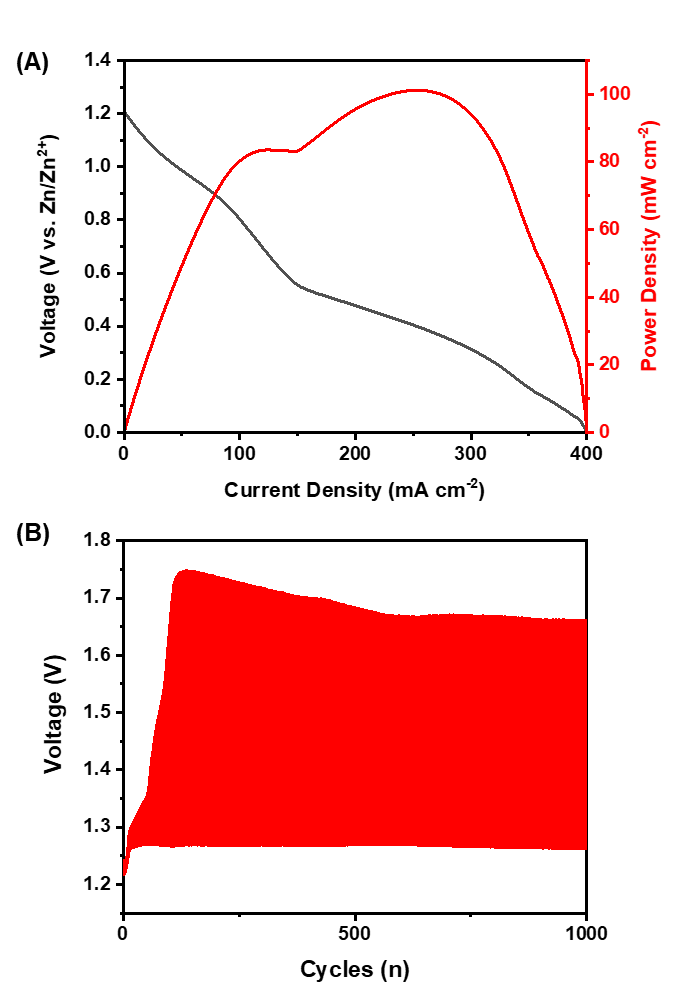
**

**Figure S13. Zinc-air battery (ZAB) performance of N-CuS under ambient air conditions. (A)** Power density curve of N-CuS obtained from the ZAB discharge profile, demonstrating a maximum power density of 101 mW cm⁻². **(B)** Galvanostatic cycling with potential limitation (GCPL) of the N-CuS ZAB over 1000 cycles with 2-minute charging/discharging duration. All other conditions are the same to that stated in the experimental section.

**
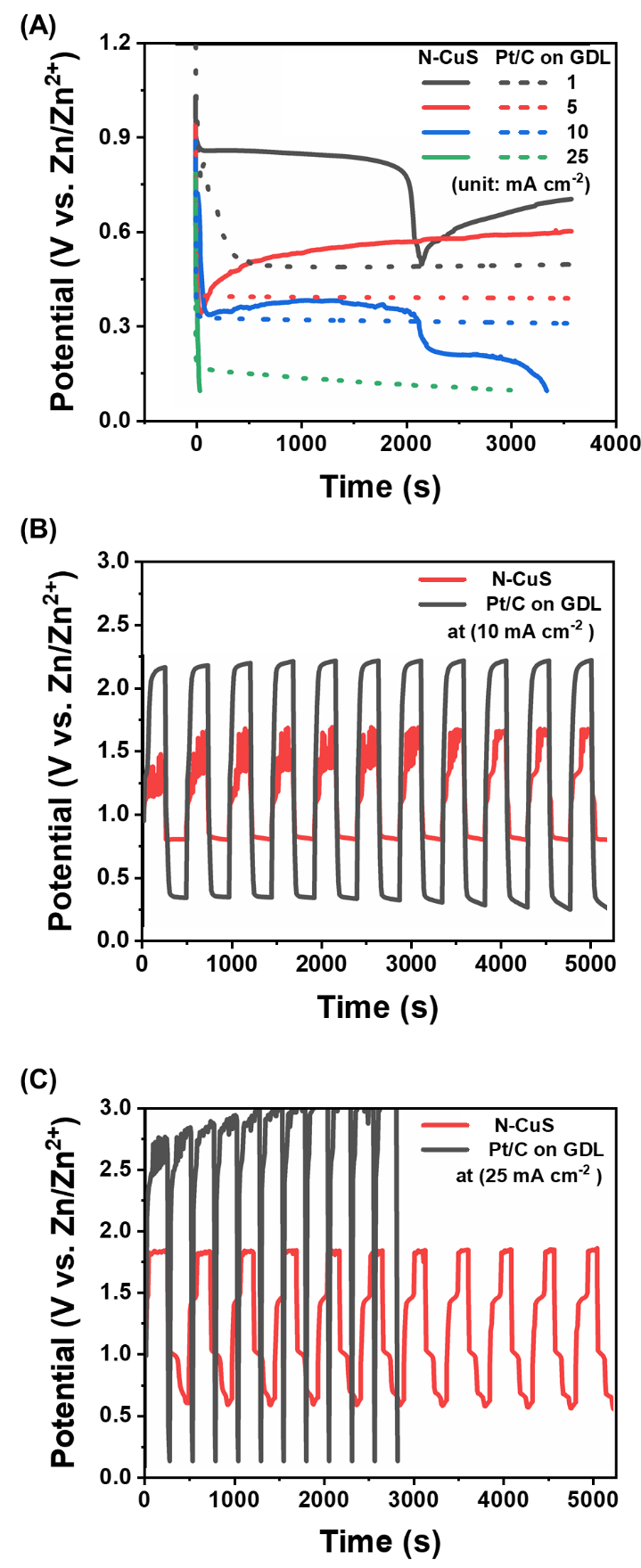
**

**Figure S14. ZAB performance of N-CuS and Pt/C on GDL compared under anaerobic conditions** (Ar Purging). **(A)** Single GCPL peak of N-CuS and Pt/C on PTFE-coated carbon paper (GDL). GCPL at **(B)** 10 mA cm^-2^ and **(C)** 25 mA cm^-2^.


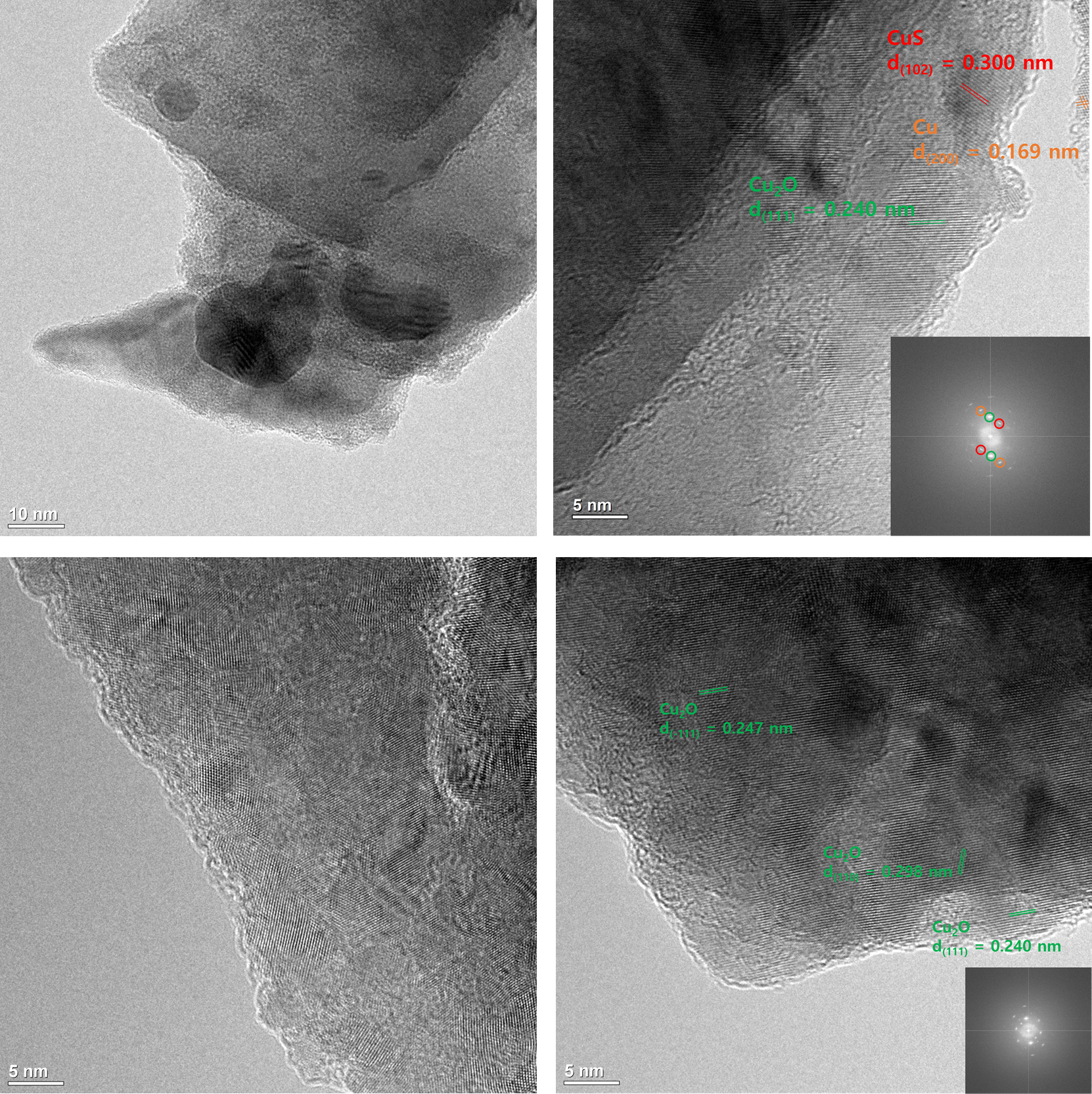


Figure S15. TEM image for N-CuS after cell cycling in anaerobic condition (Ar Purging).

**
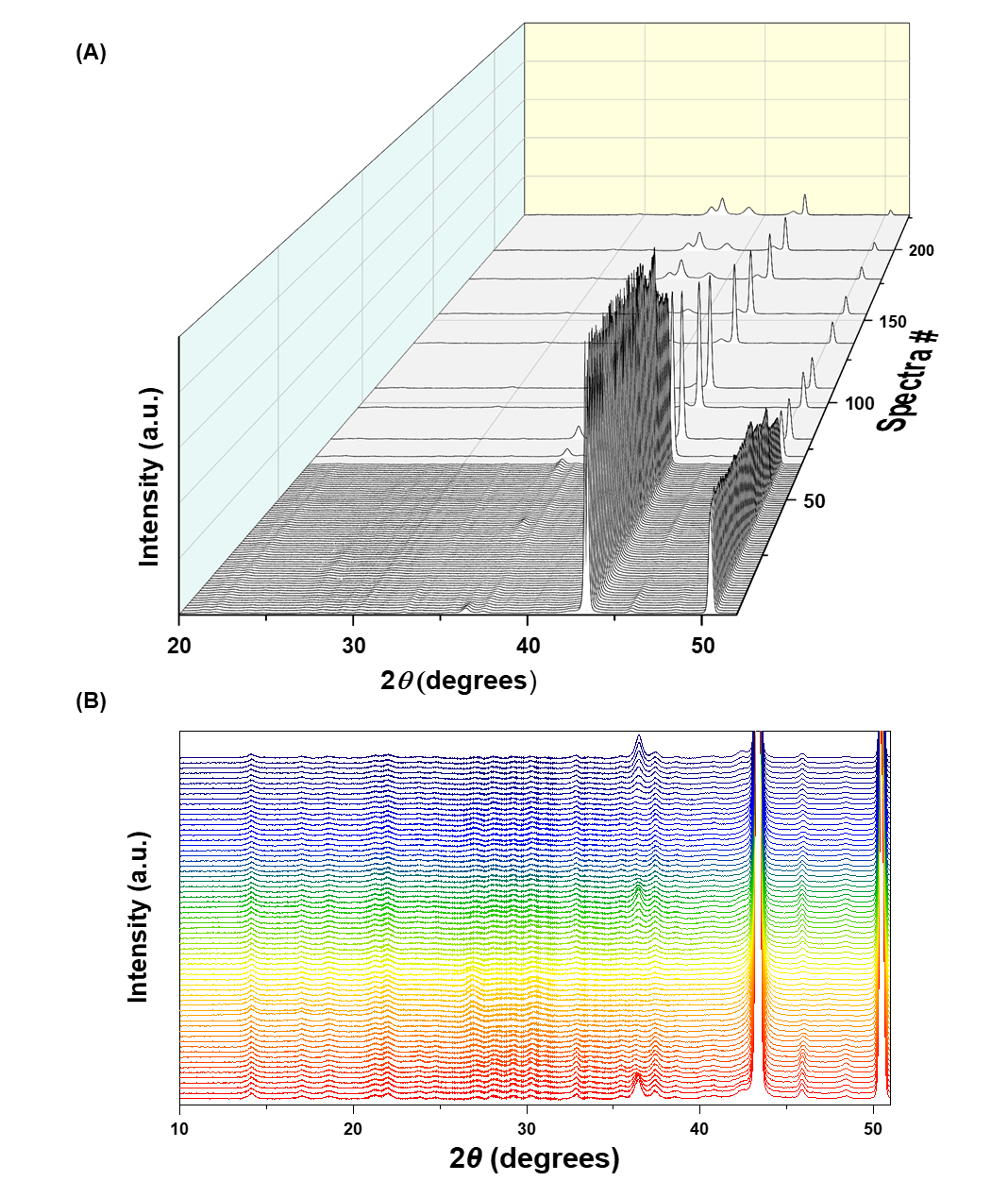
**

**Figure S16. *In-situ* X-ray diffraction (XRD) of N-CuS in anaerobic conditions**. (A) Entire spectra in a waterfall view, including XRD spectra after cell passivation. (B) Entire spectra for 1^st^ and half charge/discharge cycle.


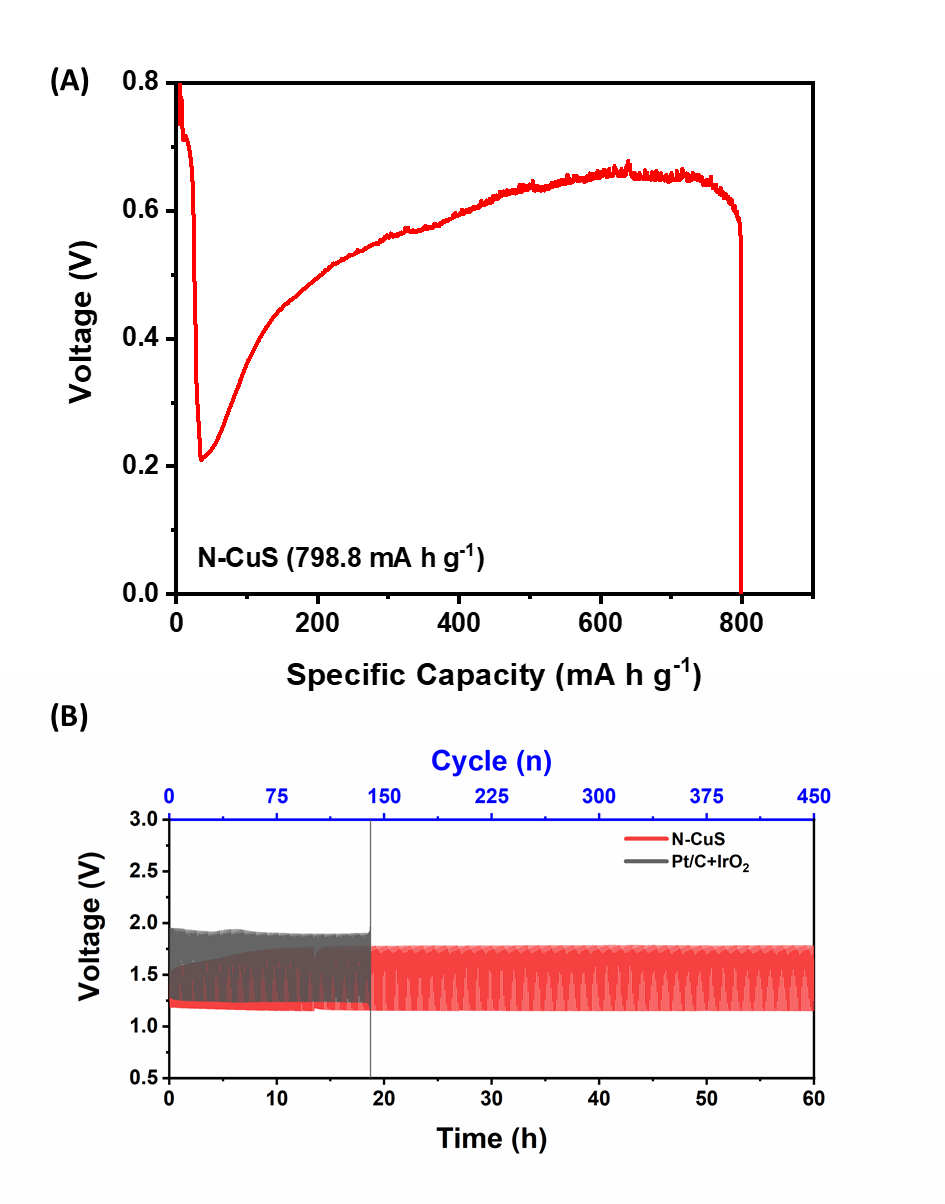


**Figure S17. Cell functionality tests. (A)** Specific capacity measurement under an anaerobic environment (Ar purging in gas purging cell) with 10 mA cm^-2^ current density. **(B)** Cell cyclability test in ZAB configuration (ambient air), using N-CuS air cathode and noble metal-air cathode (Pt/C and IrO_2_).


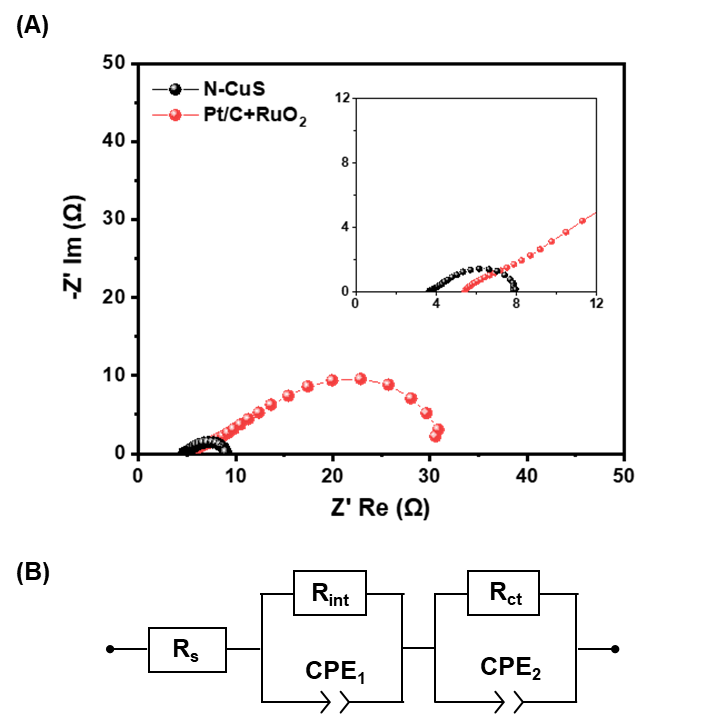


**Figure S18. EIS of ZAB configuration (ambient air) at OCV.** (A) EIS plots of N-CuS and Pt/C+RuO_2_. (inset: enlarged spectrum of N-CuS). (B) Equivalent circuit model for fitting. Where R_s_, R_int_, and R_ct_ are solution resistance, interfacial resistance, and charge transfer resistance, respectively.

**
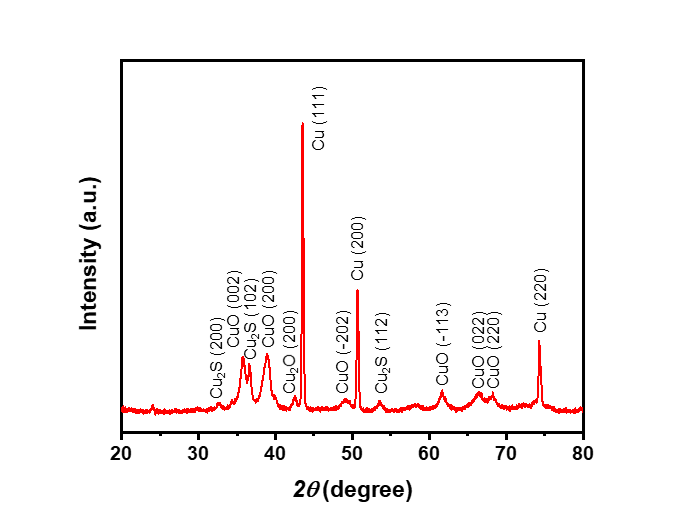
**

**Figure S19. XRD pattern of N-CuS after cycling in ZAB configuration (ambient air).**

**
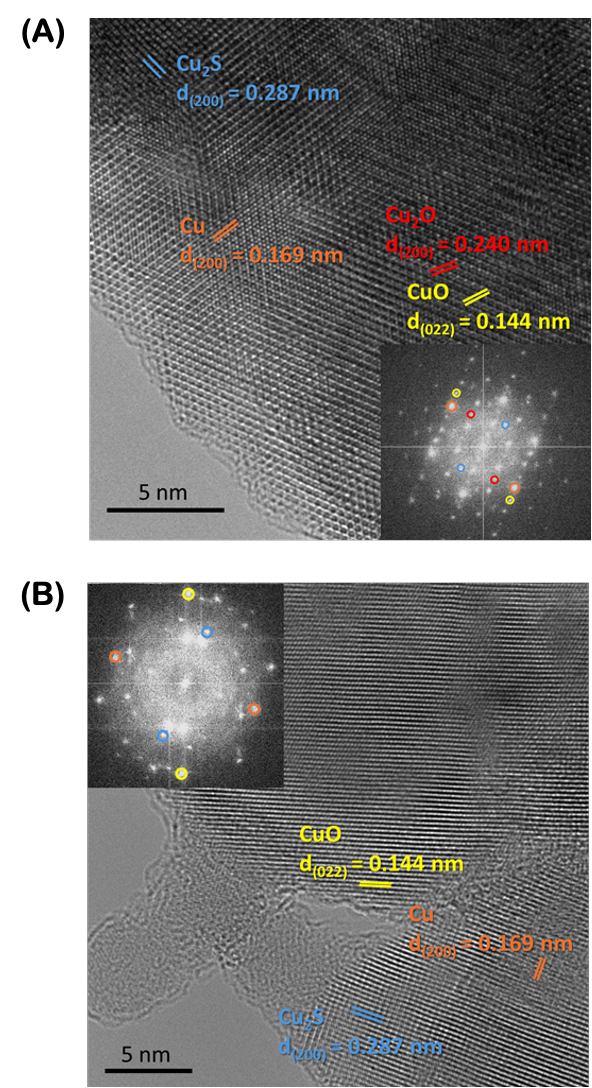
**

**Figure S20. TEM images of N-CuS after cycling in a ZAB configuration under ambient air.** Images depict cycled samples at current densities of (**A**) 10 mA cm⁻² and (**B**) 25 mA cm⁻².


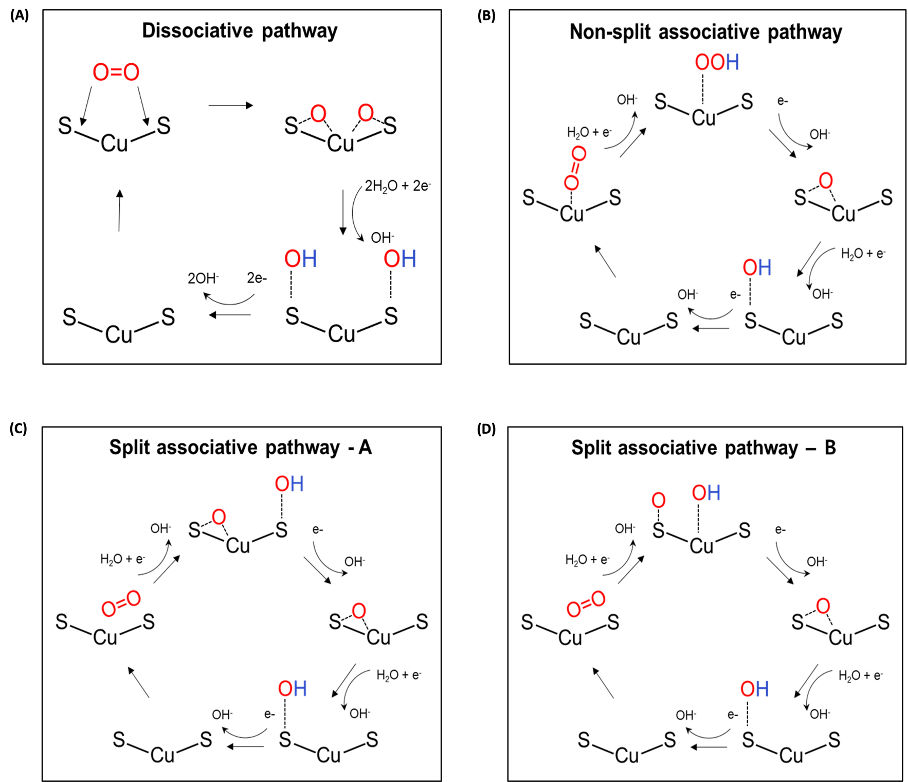


**Figure S21. Dissociative and associative ORR pathways from DFT calculations of CuS.** (A) Dissociative ORR pathway. (B) Non-split, (C) split-A, and (D) split-B associative pathways.

**
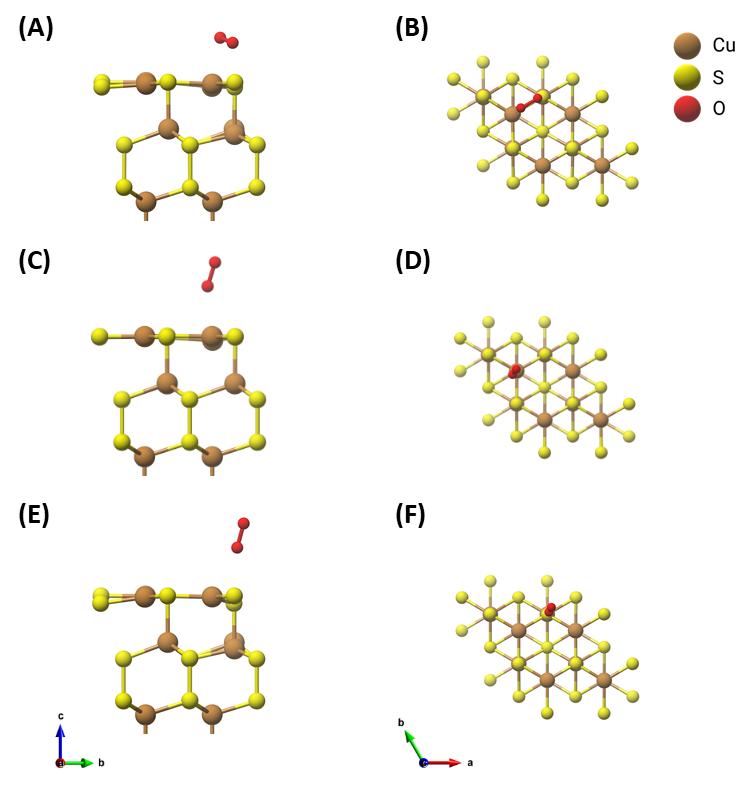
**

**Figure S22. Configurations of O_2_ adsorption on the CuS surfaces.** Side (A) and top (B) views for the side-on orientation along Cu-S. Side (C) and top (D) views for end-on orientation on the Cu atop site. Side (E) and top (F) views for end-on orientation on the S atop site.

**
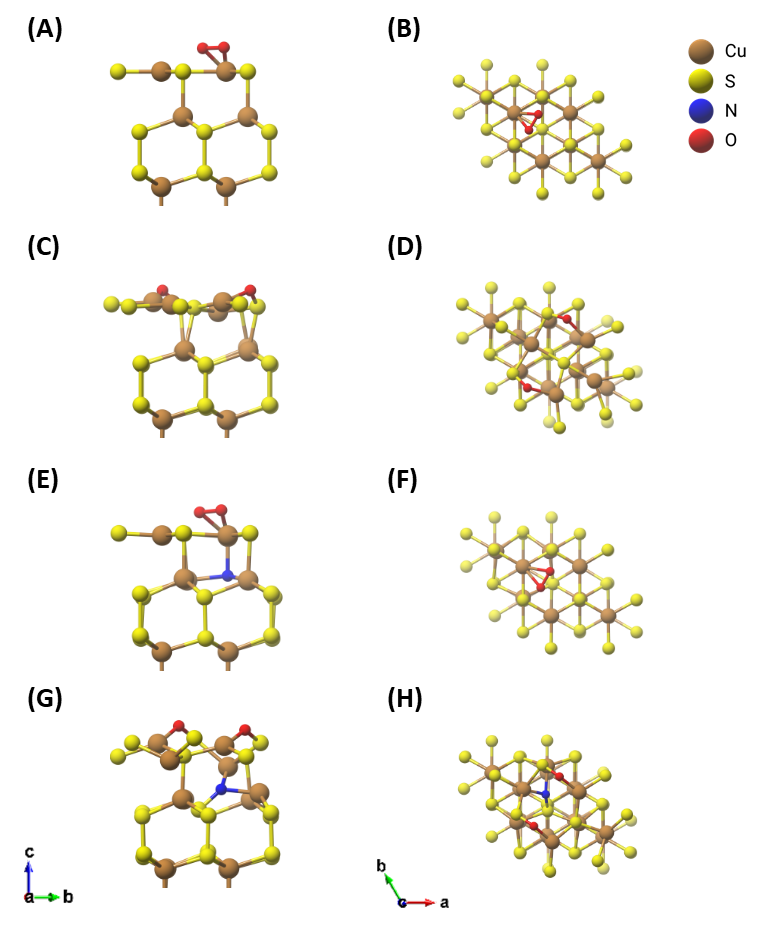
**

**Figure S23. DFT calculations showing O_2_ dissociation mechanisms on CuS and N-CuS surfaces.** (A) and (B) display side and top views of O_2_ on pristine CuS surfaces before geometry optimization, while (C) and (D) show side and top views after the dissociation into two *O atoms on the pristine surface. The (E) and (F) present the side and top views of O_2_ on N-CuS surfaces before optimization, while (G) and (H) depict the side and top views after dissociation.


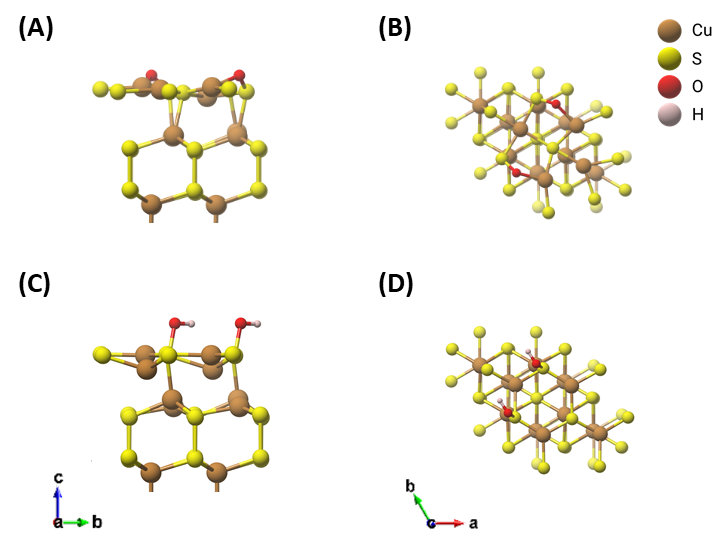


**Figure S24. Adsorbate configuration for the dissociative oxygen reduction reaction (ORR) mechanism on the CuS surface.** (A) and (B) display side and top views of the adsorbed two *O atoms, while (C) and (D) display side and top views of two adsorbed *OH intermediates on pristine surface.


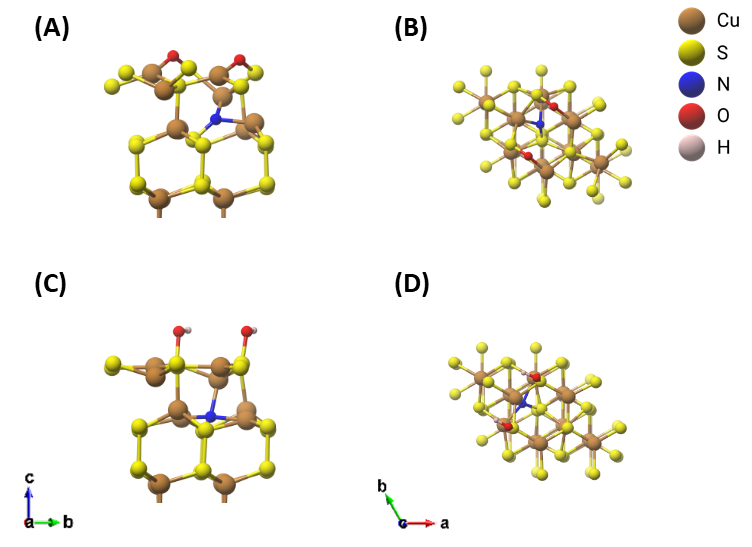


**Figure S25. Adsorbate configuration for the dissociative ORR mechanism on the N-CuS surface.** (A) and (B) display side and top views of two adsorbed *O atoms, while (C) and (D) display side and top views of two *OH intermediates on N-CuS surface.


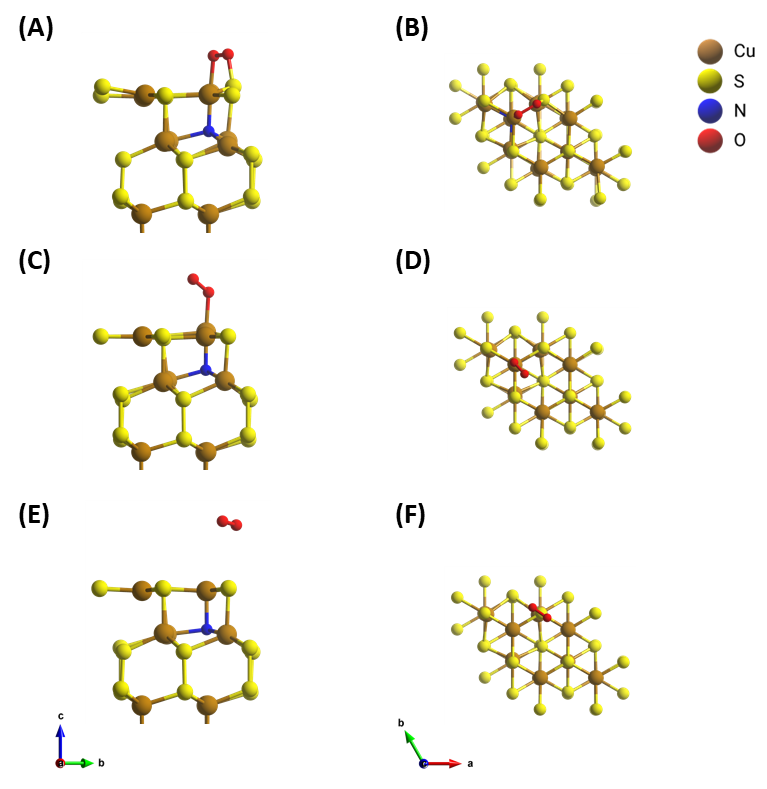


**Figure S26. Molecular configuration of O_2_ adsorption on the N-CuS surface.** (A) and (B) shows side and top views of O_2_ adsorbate with side-on orientation along Cu-S after geometric relaxation, while (C) and (D) show side and top views of O_2_ adsorbate with end-on orientation on the Cu atop site. (E) and (F) shows side and top views of O_2_ adsorbate with end-on configuration on S atop site.


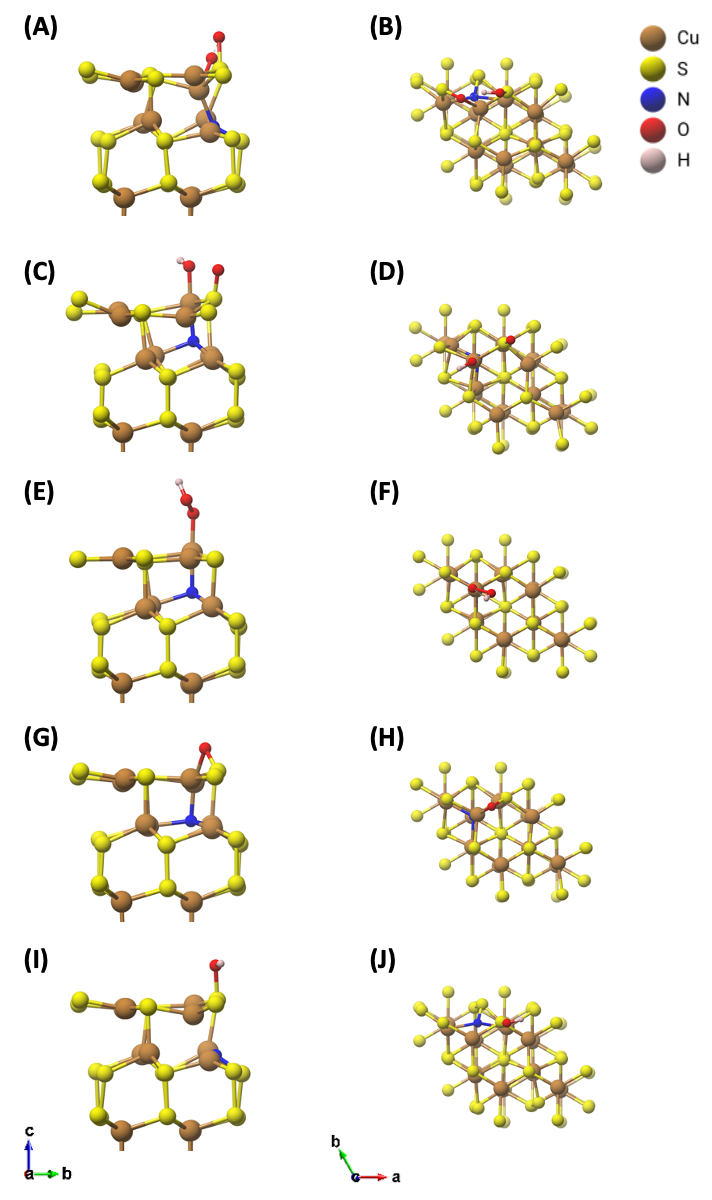


**Figure S27. Adsorbate configuration for the associative ORR mechanism on the N-CuS surface.** (A) and (B) display side and top views of *O+*OH adsorbed configuration, in which *O and *OH are placed on S and Cu, respectively (configuration A). (C) and (D) display side and top views of *O+*OH adsorbed configuration, in which *O and *OH are placed on Cu and S, respectively (configuration B). (E) and (F) display side and top views of *OOH adsorbed configuration, (G) and (H) display side and top views of *O adsorbed configuration, (I) and (J) display side and top views of *OH adsorbed configuration.

**Table S1. EXAFS fitting parameters.** All cif files are from cited literature, acquired via either the American Mineralogist Crystal Stucture Database (AMCSD) or the Crystallography Open Database (COD).

| Samples | Path | Bond Length  (Å) | Bond Disorder  (σ^2^) | R-factor  of Fit |
| --- | --- | --- | --- | --- |
| Cu Foil^[1]^ | Cu-Cu (1.1) | 2.55620 | 0.00688 | 0.0473327 |
|  | Cu-Cu (1.3) | 4.42750 | 0.01252 |  |
|  | Cu-Cu-Cu (1.1 1.2) | 4.36370 | 0.00478 |  |
|  | Cu-Cu-Cu (1.1 1.3) | 4.76990 | 0.00865 |  |
| CuO^[2]^ | Cu-O (1.1) | 1.94750 | 0.00342 | 0.0523618 |
|  | Cu-Cu (1.1) | 2.88440 | 0.00000 |  |
| CuS^[3]^ | Cu-S  (1.1) | 2.190 | 0.00837 | 0.0241224 |
| Cu_2_O^[4]^ | Cu-O  (1.1) | 1.84830 | 0.00036 | 0.0597572 |
|  | Cu-Cu  (1.1) | 3.01830 | 0.02569 |  |
| Cu_2_S^[5]^ | Cu-S (9.1) | 2.31230 | 0.00759 | 0.0122899 |
|  | Cu-S (1.1) | 2.36180 | 0.01786 |  |
|  | Cu-Cu (15.1) | 2.65500 | 0.00178 |  |
| N-CuS^[1]^ | Cu-Cu (1.1) | 2.53605 | 0.00707 | 0.0353939 |
|  | Cu-Cu (1.3) | 4.49176 | 0.01336 |  |
|  | Cu-Cu-Cu (1.1 1.2) | 3.85742 | 0.00799 |  |
|  | Cu-Cu-Cu (1.1 1.3) | 5.44208 | 0.00404 |  |
|  | Cu-N (1.1) | 4.44002 | 0.00000 |  |

**Table S2. Calculated N doping energies with different configurations shown on Figure S5D.**

| Doping configuration | | E_doping_ (eV) |
| --- | --- | --- |
| S substitutional | | -1.43 |
| Cu substitutional | | -1.70 |
| N substitutional | 1 | -1.51 |
|  | 2 | -1.45 |
|  | 3 | -1.77 |

**Table S3. Performance comparison of OER/ORR for N-CuS with recently reported catalysts.** E_ORR, 1/2_ represents the half-wave potential for ORR, and E_OER, j=10_ refers to the potential for OER at current density of 10 mA cm⁻². ΔE indicates the overall potential difference for bifunctional catalytic performance (E_OER, j=10_ – E_ORR, 1/2_). Values marked with * were separately obtained from commercial catalysts coated on Cu foams (Pt/C for ORR and RuO₂ for OER).

| Catalyst | E_ORR,1/2_  (V vs. RHE) | E_OER,j=10_  (V vs. RHE) | ΔE (V) | Reference |
| --- | --- | --- | --- | --- |
| N-CuS | 0.790 | 1.501 | 0.711 | This Work |
| CuS | 0.760 | 1.595 | 0.835 |  |
| Cu | 0.407 | 1.649 | 1.242 |  |
| Pt/C + RuO_2_ | 0.754  (0.845)* | 1.628  (1.578)* | 0.874  (0.733)* |  |
| Cu_6.81_-CoFS | 0.80 | 1.54 | 0.74 | [6] |
| (Cu, Co)_3_OS_3_@CNT-C_3_N_4_ | 0.80 | 1.66 | 0.86 | [7] |
| P,S-Co_x_O_y_/Cu@CuS NWs | 0.67 | 1.51 | 0.84 | [8] |
| Ni_3_S_2-x_Se_x_ | 0.535 | 1.611 | 1.076 | [9] |
| S-LDH/NG | 0.692 | 1.474 | 0.782 | [10] |
| Fe-Co-Ni MOF | 0.750 | 1.484 | 0.734 | [11] |

**Table S4. Atomic ratio estimated by XPS before and after electrochemical reaction.**

| Catalyst | Element (at %) | | | Assignment percentage of  peak area % | | |
| --- | --- | --- | --- | --- | --- | --- |
|  | N | S | Cu | CuS | N-CuS, Cu_2_S,Cu | CuO |
| Pristine N-CuS | 1.75 | 2.63 | 11.68 | 52 | 12 | 36 |
| After OER  (1.7 V vs. RHE) | 0.73 | 1.06 | 22.82 | 0.82 | 5.65 | 93.53 |
| After ORR  (0.2 V vs. RHE) | 1.11 | 1.44 | 15.50 | 4.75 | 25.91 | 69.34 |

**Table S5. Performance comparison of N-CuS and recently reported transition metal catalysts in Zinc-air batteries (ZABs).**

| Catalyst | Specific Capacity  (Ah kg^-1^) | Energy Density  (Wh kg^-1^) | Reference |
| --- | --- | --- | --- |
| N-CuS (Aerobic) | 788 | 916 | This Work |
| N-CuS (Anaerobic) | 799 | 449 |  |
| Pt/C + RuO_2_ | 712 | 874 | This Work |
| INF-FeCuS | 446 | N/A | [12] |
| Cu-Co/NC | 752 | N/A | [13] |
| CuCo_2_S_4_ | 331 | 424 | [14] |
| Fe_0.5_Cu_0.5_Mo_0.5_– SB-400/C | 683 | 822 | [15] |
| CoCu/N-CNS | 771 | 880 | [16] |
| Cu−N−C/F127 | 718 | N/A | [17] |
| NiFe–LDH/NG | 665 | 772 | [10] |
| Co_2_/Fe-N@CHC | 786 | N/A | [18] |
| Fe_0.5_Co@HOMNCP | 787 | N/A | [19] |

**Table S6. Calculated standard redox potentials (V) in the anaerobic condition with different calculation methods.**

| Criterion | Methodological variation | CuO → Cu_2_O | Cu_2_O → Cu |
| --- | --- | --- | --- |
| Experimental | - | 0.67 | 0.47 |
| VdW correction | None | 0.61 | 0.49 |
|  | DFT-D3  (zero damping function) | 0.54 | 0.64 |
|  | DFT-D3  (Becke-Johnson damping function) | 0.53 | 0.63 |
|  | DFT-D4 | 0.50 | 0.57 |
| No VdWcorrection | PBE+U (U_eff_ = 5) | 0.61 | 0.49 |
|  | RPBE+U (U_eff_ = 5) | 0.68 | 0.50 |
|  | HSE06 | 0.64 | 0.39 |
|  | R2SCAN | 0.45 | 0.49 |
| RPBE+U  with different U_eff_ | U_eff_ = 4 | 0.72 | 0.52 |
|  | U_eff_ = 5 | 0.68 | 0.50 |
|  | U_eff_ = 6 | 0.62 | 0.46 |

**Table S7. Comparison of atomic ratios calculated by ICP-OES and XPS.**

| Catalyst | ICP-OES/XPS (at %) | |
| --- | --- | --- |
|  | Cu | S |
| CuS | 99.28/9.11 | 0.72/1.53 |
| N-CuS | 99.34/11.68 | 0.66/2.63 |

**Supporting references**

[1] H. E. T. Swanson, E. Tatge, in *National Bureau of Standards Circular 539,* vol. 1, US Government Printing Office, Washington, DC, **1953**.

[2] L.-J. N. S. Åsbrink, *Acta Crystallogr. B* **1970**, *26*, 8.

[3] L. G. Berry, *Am. Mineral.* **1954**, *39*, 504.

[4] S. S. Hafner, S. Nagel, *Phys. Chem. Miner.* **1983**, *9*, 19.

[5] H. T. Evans, *Science* **1979**, *203*, 356.

[6] Z. Li, Q. Wang, X. Bai, M. Wang, Z. Yang, Y. Du, G. E. Sterbinsky, D. Wu, Z. Yang, H. Tian, *Energy Environ. Sci.* **2021**, *14*, 5035.

[7] X. Wang, L. Peng, N. Xu, M. Wu, Y. Wang, J. Guo, S. Sun, J. Qiao, *ACS Appl. Mater. Interfaces* **2020**, *12*, 52836.

[8] T. L. L. Doan, D. T. Tran, D. C. Nguyen, D. H. Kim, N. H. Kim, J. H. Lee, *Adv. Funct. Mater.* **2021**, *31*, 2007822.

[9] B. R. Wygant, B. A. Washington, C. N. Wright, G. A. Goenaga, T. A. Zawodzinski, T. N. Lambert, *ACS Catal.* **2023**, *13*, 9245.

[10] X. Han, N. Li, J. S. Baik, P. Xiong, Y. Kang, Q. Dou, Q. Liu, J. Y. Lee, C. S. Kim, H. S. Park, *Adv. Funct. Mater.* **2023**, *33*, 2212233.

[11] F. Shahbazi Farahani, M. S. Rahmanifar, A. Noori, M. F. El-Kady, N. Hassani, M. Neek-Amal, R. B. Kaner, M. F. Mousavi, *J. Am. Chem. Soc.* **2022**, *144*, 3411.

[12] J. Dang, M. Yin, D. Pan, Z. Tian, G. Chen, J. Zou, H. Miao, Q. Wang, J. Yuan, *Chem. Eng. J.* **2023**, *457*, 141357.

[13] Z. Li, S. Ji, C. Wang, H. Liu, L. Leng, L. Du, J. Gao, M. Qiao, J. H. Horton, Y. Wang, *Adv. Mater.* **2023**, *35*, e2300905.

[14] Y. Li, J. Yin, L. An, M. Lu, K. Sun, Y.-Q. Zhao, F. Cheng, P. Xi, *Nanoscale* **2018**, *10*, 6581.

[15] T. H. Chiang, Y.-S. Chen, *Catal. Sci. Technol.* **2023**, *13*, 3505.

[16] J. Kuang, Y. Shen, Y. Zhang, J. Yao, J. Du, S. Yang, S. Zhang, Y. Fang, X. Cai, *Small* **2023**, *19*, 2207413.

[17] W.-J. Niu, W.-W. Zhao, Y.-Y. Yan, C.-Y. Cai, B.-X. Yu, R.-J. Li, *J. Colloid Interface Sci.* **2024**, *675*, 989.

[18] Z. Wang, X. Jin, C. Zhu, Y. Liu, H. Tan, R. Ku, Y. Zhang, L. Zhou, Z. Liu, S.-J. Hwang, H. J. Fan, *Adv. Mater.* **2021**, *33*, 2104718.

[19] W. Li, B. Liu, D. Liu, P. Guo, J. Liu, R. Wang, Y. Guo, X. Tu, H. Pan, D. Sun, F. Fang, R. Wu, *Adv. Mater.* **2022**, *34*, 2109605.
